# Sesquiterpene Backbones Generated by Sesquiterpene Cyclases: Formation of *iso*-Caryolan-1-ol and an Isoclovane

**DOI:** 10.1021/acs.orglett.3c03383

**Published:** 2023-11-27

**Authors:** Henry Struwe, Finn Schrödter, Hanke Spinck, Andreas Kirschning

**Affiliations:** Institute of Organic Chemistry, Leibniz University Hannover, Schneiderberg 1B, 30167 Hannover, Germany

## Abstract

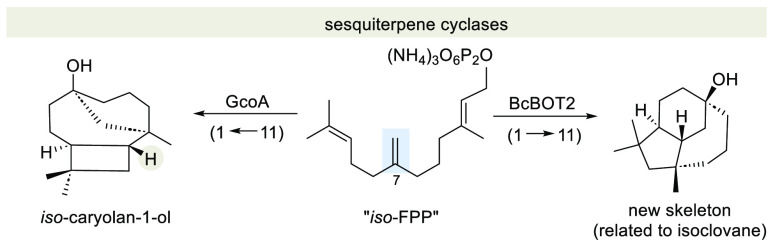

New sesquiterpene backbones are accessible after incubation of caryolan-synthase (GcoA) and presilphiperfolan-8-β-ol synthase (BcBOT2) with a non-natural farnesyldiphosphate in which the central olefinic double bond is isomerized toward the methyl group. Two newly formed sesquiterpenoids are reported, a constitutional isomer of caryolan-1-ol (**3**), which we name *iso*-caryolan-1-ol (**17**), and the first terpenoid based on the isoclovane ring skeleton generated enzymatically thus far.

Tricyclic sesquiterpenes can be considered a subset of C15 sesquiterpenes. Typical examples are presilphiperfolan-8-β-ol (**2**) and (+)-caryolan-1-ol (**3**). Biosynthetically, the tricyclic frameworks in sesquiterpenes result from the fact that the operating sesquiterpene cyclases (STCs) convert farnesyl pyrophosphate (**1**) in such a way that all three olefinic double bonds are involved in the cationic cascade reaction. If the final carbocation is intercepted by water, then no olefinic double bond is found in the final cyclization product and an alcohol functional group is present instead, as is the case in sesquiterpenes **2** and **3** (Scheme 1). The terpene cyclases that are responsible for the formation of the two sesquiterpenes are the fungal presilphiperfolan-8-β-ol synthase (BcBOT2) from *Botrytis cinerea*(1) and the bacterial caryolan synthase (GcoA) from *Streptomyces griseus*.^2^

**Scheme 1:**
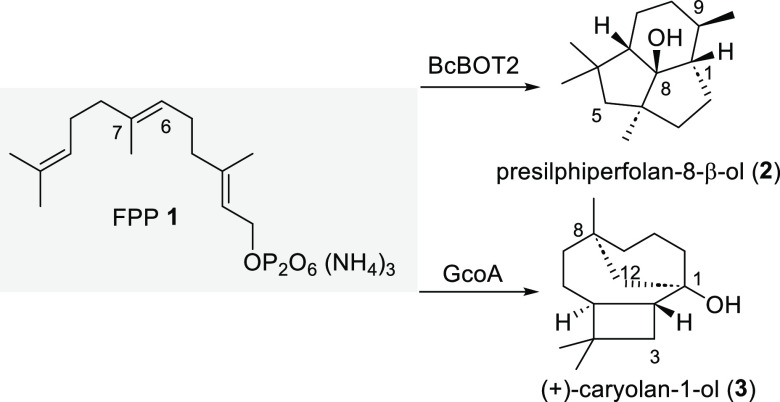
Structures of Farnesyl Pyrophosphate (FPP; **1**), Presilphiperfolan-8-β-ol (**2**), and (+)-Caryolan-1-ol (**3**) Numbering for **2** according to ref (1b). Numbering for **3** according to ref (2).

In recent years our group^3,4^ and others^5^ demonstrated that sesquiterpene cyclases show a surprisingly pronounced promiscuity toward chemically modified farnesyl pyrophosphate derivatives **4**–**8** (Figure 1), and details of the resulting biotransformation products formed by BcBOT2 can be found in the literature.^3,4^ This paves the way to enlarge the structural space and structural diversity of terpenes and in selected cases^6^ allows additional information to be provided on the proposed mechanisms with FPP **1**.^5^ Recently, diterpene cyclases have also been probed for transformations with unnatural geranylgeranyl pyrophosphate (GGPP) derivatives.^7^

**Figure 1:**
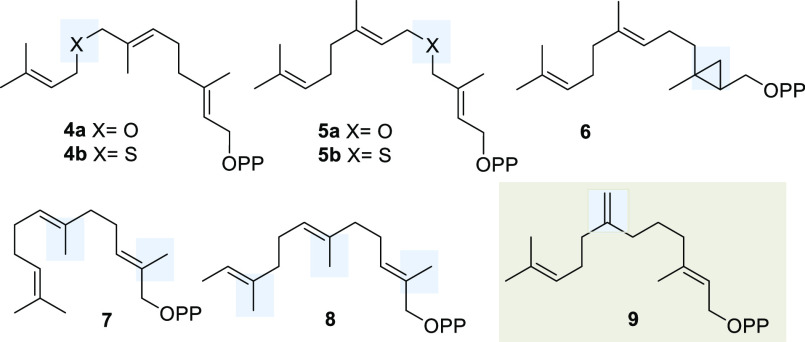
Structures of FPP derivatives **4**–**8** and FPP derivative **9** studied in this work (structural deviations from **1** are labeled in light blue).

For the present work, we selected 7-methylene farnesylpyrophosphate (**9**) as the FPP derivative. Allemann and co-workers first introduced this derivative^5c^ by treating it with aristolochene synthase (PR-AS) from *Penicillium roquefortii*. This STC does not generate a tricyclic product, and indeed it behaved like FPP, also yielding the bicyclic aristolochene when exposed to **9**. However, we hypothesized that structural changes around the central olefinic double bond (C6=C7) should have an impact on the cationic cascades promoted by STCs, which generate tricyclic sesquiterpene backbones. This stems from the fact that the central olefinic double bond is involved in cyclization at a later stage of the cationic cascade of such STCs, so that mechanistically the first steps supposedly would not be affected.

Our synthesis of 7-methylene farnesylpyrophosphate (**9**) markedly differs from the one reported by Allemann^5c^ and commenced from hex-5-yn-1-ol **10** (Scheme 2). After *O*-silylation, the alkyne was subjected to a Zr-catalyzed carboalumination. The organometallic intermediate was then trapped with formaldehyde, which yielded allyl alcohol **11**.^8a,8b,9^ After a series of functional group manipulations, followed by Swern oxidation, the resulting aldehyde **12** was homoallylated with lithiated bromide **15** and finally the resulting alcohol was oxidized to furnish ketone **13**.

**Scheme 2:**
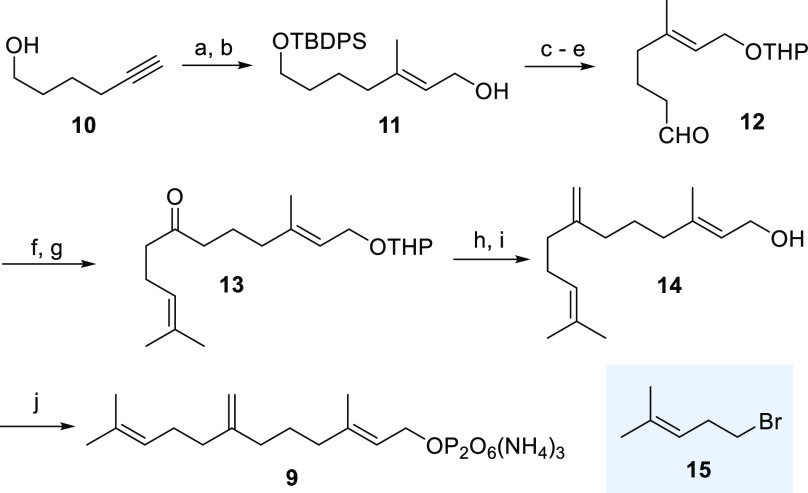
Synthesis of FPP Derivative **9** Reagents and conditions: (a) TBDPSCl, imidazole, CH_2_Cl_2_, 0 °C to rt, 95%; (b) ZrCp_2_Cl_2_, H_2_O, AlMe_3_, CH_2_O, CH_2_Cl_2_, 0 °C to rt, 82%; (c) DHP, PPTS, CH_2_Cl_2_, 0 °C to rt, 89%; (d) TBAF, THF, 0 °C to rt; e) DMSO, (ClCO)_2_, Et_3_N, CH_2_Cl_2_, −78 °C to rt, 91%; (f) *t*-BuLi, bromide **15** Et_2_O, −78 °C to rt, 61%; (g) DMSO, (ClCO)_2_, Et_3_N, CH_2_Cl_2_, −78 °C to rt, 83%; (h) MePPh_3_Br, *n*-BuLi, THF, 0 °C to rt, 81%; (i) PPTS, EtOH, 50 °C, 93%; j) DMS, NCS, CH_2_Cl_2_, 0 °C to rt, workup then ((*n*-Bu)_4_N)_3_P_2_O_7_H, MeCN, rt, 55%. DHP = dihydropyrane, PPTS = *p*-toluenesulfonic acid, TBAF = tetra-*n*-butylammonium fluoride, DMS = dimethyl sulfide, and NCS = *N*-chlorosuccinimide.

The synthetic sequence toward FPP derivative **9** was terminated by Wittig olefination, removal of the THP protection (to yield allyl alcohol **14**), and introduction of the diphosphate moiety via chlorination according to the protocol developed by Poulter et al.^10^

Next, the STCs BcBOT2 and GcoA were expressed in *Escherichia coli* as reported before^3a^ and detailed in the SI. To determine enzyme activity and substrate tolerance, *in vitro* enzyme assays were conducted with both FPP **1** and derivative **9** (500 μL scale, 0.1 g/L BcBOT2 or GcoA, 37 °C for **9**, 30 °C, 12 h), which yielded new major transformation products as judged by GC-MS analysis (R_I_ = 1678 and *m*/*z* 222 for **16**, R_I_ = 1531 and *m*/*z* 222 for **17**).

A close inspection of the GC-MS chromatogram reveals that the incubation of BcBOT2 with 7-methylene farnesylpyrophosphate (**9**) furnished several other products, as listed in Table S1 (Supporting Information). However, the amounts were too small to isolate sufficient amounts for structure elucidation. Transformation of FPP derivative **9** by GcoA mainly gave cyclization product **17**, and all other GC peaks had relative abundances of <1% compared to the main product. The relative stereochemistry was elucidated by determining selected nuclear Overhauser effects, which are summarized in Figure 2.

**Figure 2:**
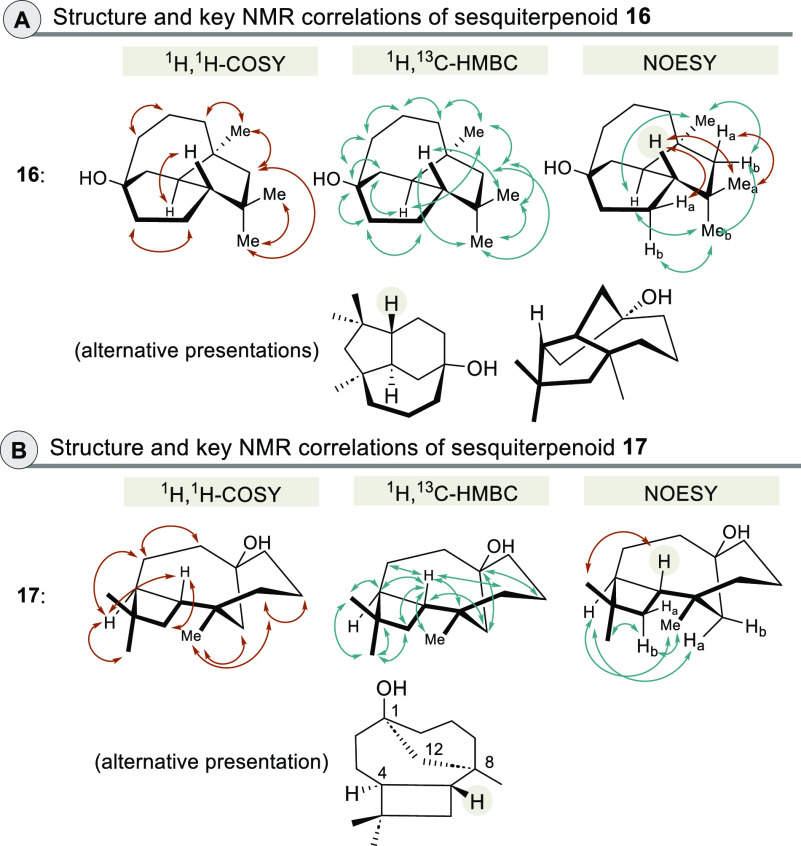
New sesquiterpene alcohols **16** and **17** (different views) and key NMR correlations for structural elucidations (colors of arrows used for NOESY correlations refer to the α- and β-faces).

The upscaling provided sufficient amounts of material (1 mg each) to characterize the new main sesquiterpenoids **16** and **17**. The characterization of the new terpenoids was based on different NMR spectroscopic techniques including H,H–COSY, HMBC, and HSQC, while the relative stereochemistry was elucidated by determining selected nuclear Overhauser effects. The key correlations are summarized in Scheme 3. Further confirmation of the structural proposal for sesquiterpenoid **17** was also obtained from the comparison of selected chemical shifts (δ) in the ^13^C NMR spectrum with those published for (+)-caryolan-1-ol (**3**).^2^ We found that the ^13^C NMR signals of the stereogenic centers in the cyclobutane ring are δ 40.4 and 46.5 ppm, and the corresponding values in sesquiterpene **3** were reported as δ 39.5 and 44.7 ppm, respectively. The values determined for the methylene bridge are also diagnostic (**17**, δ 50.5 ppm; **3**, δ 48.7 ppm), as well as those for the quaternary C atom carrying the geminal methyl groups (**17**, δ 34.6 ppm; **3**, δ = 35.0 ppm) showing similar values.

**Scheme 3:**
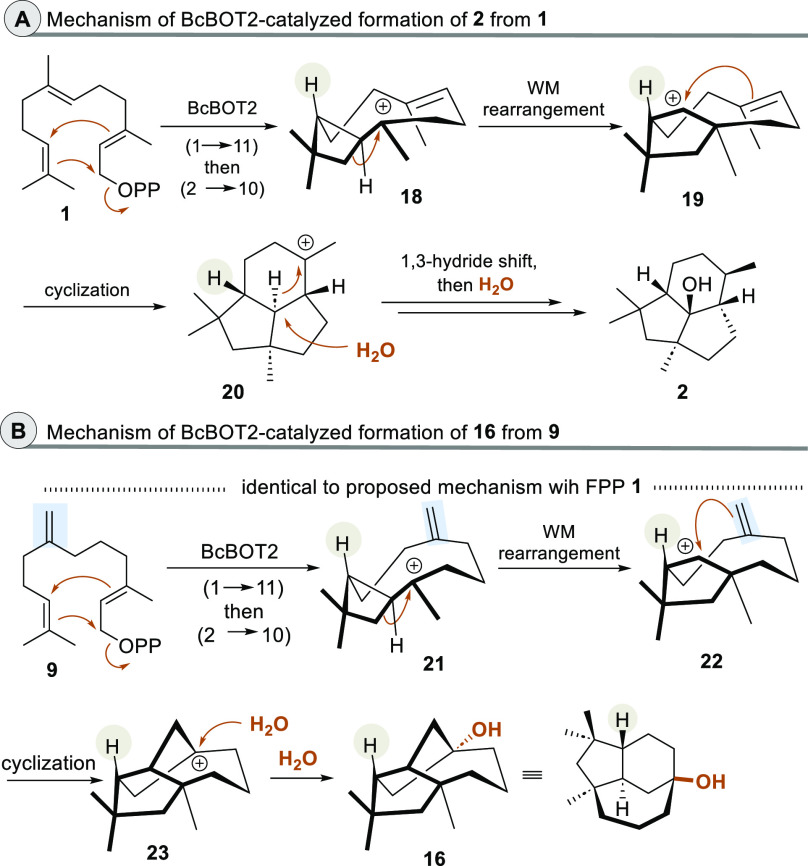
Comparison of Proposed Mechanisms toward **2** and **16** by BcBOT2 The stereogenic center with the H substituent that serves as a reference point for the absolute stereochemistry is labelled.

Mechanistic considerations for the formation of the new terpenoids **16** and **17** should be guided by the proposed mechanisms discussed for the corresponding biotransformations with FPP **1** as a substrate. The biosynthesis of presilphiperfolan-8-β-ol (**2**) from FPP (**1**)^1^ is initiated by a 1 → 11 ring closure and formation of the humulyl cation, which is followed by a second 2 → 10 cyclization to yield the methyl cyclobutyl cation **18** (Scheme 3 A). After ring expansion, the cyclopentyl cation **19** is formed which initiates a third ring closing step leading to cation **20**. From there, a 1,3-hydride shift followed by addition of water leads to **2**. Theoretical studies suggest that the Wagner-Meerwein rearrangement along with the third cyclization (**18** → **20**) may proceed via a transition state structure more closely resembling a nonclassical carbocation.^11^

For 7-methylene farnesylpyrophosphate (**9**) and the formation of **16** by BcBOT2, one can suggest that mechanistically the first steps (**9** → **22**) are identical to those (**1** → **19**) proposed for presilphiperfolan-8-β-ol (**2**) (Scheme 3 B), which matches our considerations when we decided to choose FPP derivative **9** as a suitable substrate to create new tricyclic sesquiterpene skeletons. From there, however, the route deviates in that the remaining alkene in **22** initiates cyclization via the “*exo*-located” carbon atom to form the tricyclic bridged intermediate **23** to which water is added to form sesquiterpenoid **16**, formally bearing a bicyclo [4.3.1] decane core.

The mechanistic considerations of the formation of terpenoid **16** also provide information on the likely absolute stereochemistry. The stereogenic center with the substituted hydrogen atom, labeled in gray, is formed during cyclobutane formation and remains unaltered for both mechanistic pathways (leading to **2** and **16**, respectively). The relative and the absolute configurations of presilphiperfolan-8-β-ol (**2**) were unequivocally determined spectroscopically,^12^ by derivatization to silphiperfol-6-enes,^12^ by X-ray crystallographic analysis of the *p*-nitrobenzoate,^1b^ and recently by total synthesis.^13^ Consequently, we assume that the stereogenic center is (*R*)-configured in the tricyclic product **16**.^3a^

The proposed biosynthesis of caryolan-1-ol (**3**)^2^ by GcoA is also initiated by a 1 → 11 ring closure followed by a 2 → 10 cyclization (Scheme 4 A). The intermediate methylcyclobutyl cation **18** shown in Scheme 3 undergoes deprotonation. The resulting diene **24** was proposed to be a neutral intermediate. From there, reprotonation of the alkene at C6–C7 and nucleophilic attack of the *exo*-alkene induces the final cyclization and formation of the tertiary carbocation **25** that leads to **3** after the addition of water.

**Scheme 4:**
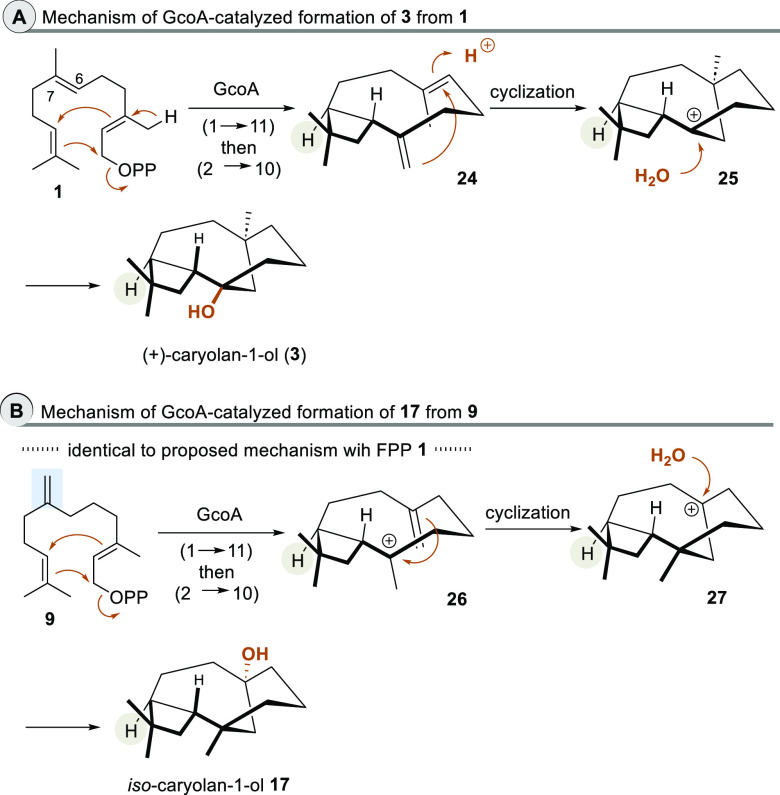
Comparison of Proposed Mechanisms towards **3** and **17** by GcoA The stereogenic center with the H substituent that serves as a reference point for the absolute stereochemistry is labelled.

The first steps (**9** → **26**) in the biotransformation of 7-methylene farnesylpyrophosphate (**9**) to terpenoid **17** by GcoA are mechanistically identical to those (**1** → **18**) proposed for caryolan-1-ol (**3**) (Scheme 4 B). This cation does not undergo deprotonation to a diene intermediate similar to **24** but directly reacts with the “*exo*-positioned” olefinic double bond to yield the cyclization product **27** that furnishes *iso*-caryolan-1-ol (**17**) after the addition of water.

The determination of the absolute configuration of caryolan-1-ol (**3**) from *S. griseus* had some hurdles.^2^ Ohnishi and co-workers found that when GcoA was expressed in *Streptomyces lividans*, (+)-caryolan-1-ol (**3**) was isolated as judged by spectroscopic analyses using chiral GC. (+)-Caryolan-1-ol (**3**) was also detected in the crude cell lysate of wild-type *S. griseus* but not in the GcoA knockout mutant. From these observations they concluded that GcoA is a genuine (+)-caryolan-1-ol synthase. As for the formation of terpenoid **16**, mechanistic considerations can also suggest the likely absolute stereochemistry of *iso*-caryolan-1-ol (**17**). Here, the stereogenic center with the H substituent labeled in gray is formed during cyclobutane formation. Its absolute configuration does not change for both cationic cascades leading to **3** and **17**.

How can the backbones of new sesquiterpenoids **16** and **17** be related to the world of tricyclic sesquiterpenes? In the case of terpene alcohol **16**, the relationship to caryolan-1-ol (**3**) can be readily and easily established. The methyl group and the alcohol have simply exchanged their positions (Figure 3, top) so that we suggest naming this new sesquiterpenoid *iso*-caryolan-1-ol (**17**). The carbon skeleton of **17** relates to the rare isoclovane skeleton, and isoclovene **26** is the most prominent member (Figure 3, bottom). Its skeleton is isomeric to clovene **27**, which is present at low concentration during Shiraz grape ripening.^14^

**Figure 3:**
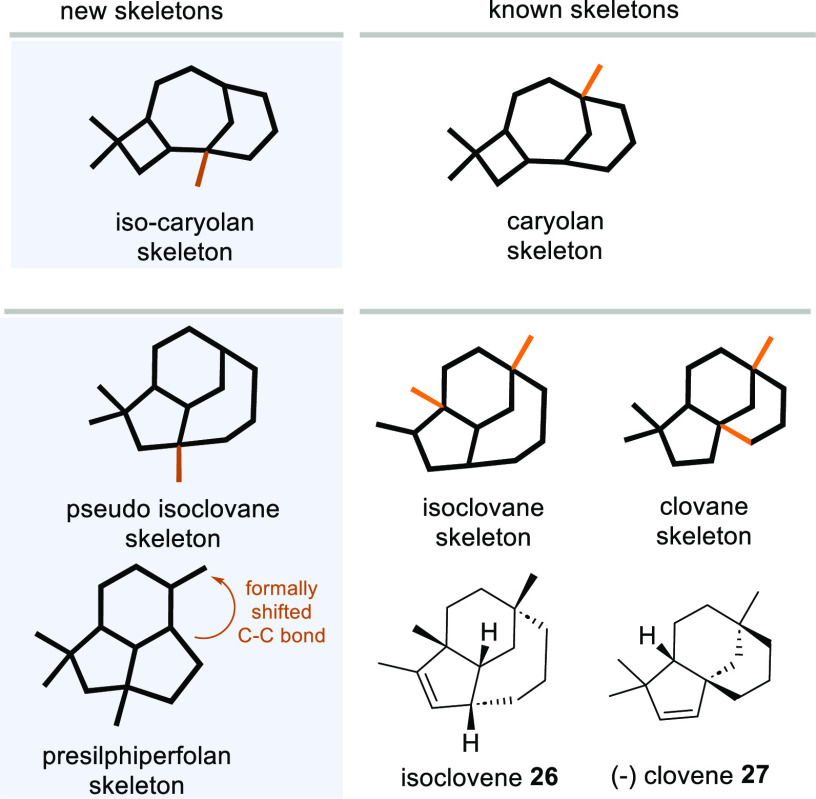
New skeletons and their relationship to related backbones of caryolan, isoclovane, and clovane (the differences in constitutions are marked in orange), structures of isoclovene (**26**) and (−)-clovene (**27**), and the relationship between the new clovane skeleton and the natural presilphiperfolan backbone formed by BcBOT2.

The tricyclic systems present in **17** and **26** are identical except that the positions where the methyl groups are bound to differ (highlighted in orange in Figure 3). Isoclovene **26** has not been isolated from natural sources so far, nor has a sesquiterpene cyclase been reported that is able to generate its skeleton from FPP **1**. In fact, it was chemically manifested as the major product formed from caryolan-1-ol (**3**) under acidic conditions via a complex cationic cascade after protonation of the hydroxy group in **3**.^15−17^ Thus, we provide the first example of the enzyme-catalyzed formation of the tricyclic isoclovane backbone in the present using a non-natural FPP derivative.

In summary, we demonstrated that small changes in the positioning of the central double bond in FPP lead to new tricyclic sesquiterpene backbones. In the case of the sesquiterpene cyclase GcoA, an isomer of the natural (+)-caryolan-1-ol (**3**) is generated in which one methyl group and the alcohol functionality have switched positions. In the case of BcBOT2, the shift of the central olefinic double bond toward the methyl group yields a new sesquiterpene alcohol that is based on a carbon skeleton unknown for the natural terpenome.^18^ This work is further proof that STCs exhibit a high degree of promiscuity toward unnatural FPP derivatives, and that the structural diversity of natural terpenes can be significantly increased via the chemoenzymatic approach reported here.

## Data Availability

The data underlying this study are available in the published article and its Supporting Information

## Supporting Information Available

Detailed procedures and spectral data (PDF)
