# Supplementary material for: Sesquiterpene Backbones Generated by Sesquiterpene Cyclases: Formation of iso-Caryolan-1-ol and an Isoclovane
Source: Org Lett. 2023 Nov 27;25(48):8575–9. doi: 10.1021/acs.orglett.3c03383 (PMC10714441; doi:10.1021/acs.orglett.3c03383)
Supplement: Supplementary file 1 — ol3c03383_si_001.pdf [file ol3c03383_si_001.pdf]

Sesquiterpene backbones generated by sesquiterpene cyclases – formation of *iso*-caryolan-1-ol and an isoclovane

Henry Struwe, Finn Schrödter, Hanke Spinck, and Andreas Kirschning*

Institute of Organic Chemistry, Leibniz University Hannover, Schneiderberg 1B, 30167 Hannover, Germany

**1.**	**Materials and methods**	page S1
1.1	General information	page S1
1.2	Chemical synthesis	page S3
1.3	Microbiological methods and biotransformations	page S8
1.4	GC-MS data	page S11
1.5	Structure elucidation of product **16**	page S13
1.6	Structure elucidation of product **17**	page S19
2.	**Copies of NMR spectra**	page S25
**3.**	**References (supporting information)**	page S38

1. Materials and methods

1.1 General information

The experimental procedures described below, in which water is neither a reactant nor used as a solvent, were carried out in pre-dried flasks under an inert gas atmosphere. To ensure homogeneity, the reaction mixtures were stirred with a magnetic stirrer. The temperatures indicated refer to the bath vessels used. Temperatures of −78 °C were achieved by an acetone-dry ice mixture and 0 °C by a mixture of water and ice. The room temperature does not refer to a specific value, but varies with the ambient temperature. For varying temperatures oil baths, NaCl-ice mixtures or a cryostat were used.

Unless stated otherwise, dry solvents were used for each reaction in which a pre-dried flask was used. Tetrahydrofuran, dichloromethane, *N,N*-dimethylformamide, acetonitrile and diethyl ether were obtained as dry solvents from a “Braun” solvent purification system. The deuterated solvents used for NMR spectroscopy were obtained from Deutero GmbH

Column chromatography was performed using silica gel obtained from Macherey-Nagel (particle size 40-63 µm). During chromatography pressure was applied and the used solvents/mixtures are listed in the corresponding experimental procedure. To perform TLC analysis pre-coated TLC sheets ALUGRAM® Xtra SIL G/UV_{254} foil from Macherey-Nagel (layer: 0.20 mm silica gel 60 with fluorescent indicator UV_{254}) were used. As stain solutions reagents containing vanillin or $KMnO_4$ were used. In selected cases UV light (λ = 254 nm/ 366 nm) was employed to identify TLC spots. The R_f values given are subject to certain inaccuracies due to measurement limitations, but can be used for initial orientation. Volumes given as a sum refer to the amount of solvent used for the first solution and the additional solvent used for rinsing the glassware (e.g. solvent (2 mL + 2 mL)).

NMR data (^{1}H, ^{13}C, ^{31}P, DEPT135, 2D-NMR) were recorded on the following spectrometers: Bruker AVANCE I ($\nu_L(^{1}H)$=400 MHz) equipped with a DUL probe, Bruker AVANCE III HD ($\nu_L(^{1}H)$=400 MHz) equipped with either a PRODIGY BBFO or BBO probe, Bruker AVANCE III HD ($\nu_L(^{1}H)$=500 MHz) equipped with a TCI cryoprobe, and Bruker AVANCE NEO ($\nu_L(^{1}H)$=600 MHz) equipped with DUL cryoprobe. All probes are equipped with z-Gradient coils. The deuterated solvents are given in the respective procedures ($CDCl_3$, C_6D_6, D_2O). The analysis was performed using the Bruker Topspin® software. The residual solvent signal of the deuterated solvents was used to calibrate the chemical shift scale of the NMR spectra. Chemical shifts δ are given in ppm, J coupling constants are given in Hz and were determined manually or with appropriate software functions. The abbreviations used for multiplicities are s (singlet), d (doublet), t (triplet), q (quartet), qi (quintet), and m (multiplet). If a signal shows a main coupling that can be identified e.g. as a triplet and additionally one of higher order, only the coupling that can be clearly identified will be described in the analytical data. If a particular signal cannot be unambiguously assigned to a particular position, e.g. because signals overlap, the indices in question are separated by "/" to indicate that there are several possibilities.

HR-ESI-MS analysis was performed with a LCT premier by Waters with a lock spray dual ion source. GC-EI-MS was measured on a GC-MS HP MSD-5973 / GC-6890 (column: Optima WAX) and an Agilent 5977B GC/MSD with 7890B GC-system (column: Optima5HT). HR-CI-MS was performed with a HP 6890 Series GC-system by Hewlett Packard.

Ion exchange of tetra-*n*-butylammonium to ammonium cations was performed using DOWEX®50WX8 resin as column material. For this purpose, the resin was rinsed with an aqueous HCl solution (3 M) until the eluent was acidic (pH paper). It was then rinsed with H_2O to a near neutral pH, with NH_3 (6%) to an alkaline pH, and finally with ion exchange buffer (980 mL H_2O, 20 mL *i*-PrOH, 2 g NH_4HCO_3) to a pH of 8 to 9. The residues collected from the reactions were then loaded onto the column and eluted with ion exchange buffer. The product-containing fractions, analyzed by vanillin- or $KMnO_4$-TLC staining, were combined and the solvent was removed *in vacuo*. The residue was diluted in an aqueous NH_4HCO_3 solution (0.05 M) and freeze-dried. To remove the inorganic ammonium pyrophosphate salt, the product was dissolved in an aqueous NH_4HCO_3 solution (0.05 M, 2 mL) and mixed with MeCN/*i*-PrOH (1/1, 8 mL). After centrifugation (5000 rpm, 10 min, 4 °C), the liquid was collected and the procedure was repeated with the solid obtained. After removal of the solvent in vacuo, the residue was dissolved in an aqueous NH_4HCO_3 solution (0.05 M) and freeze-dried. The product was stored at temperatures between −70 °C and −80 °C. The diphosphate salts were analyzed not only by ^{1}H- and ^{13}C- but additionally by ^{31}P- NMR spectroscopy. HRMS measurements and determination of R_f values could not be performed on these salts.

Experiments with living microorganisms were performed in either S1 or S2 laboratories. Sterile work was performed using a Thermos Scientific laminar flow cabinet (type 2020). Optical density (OD600) was measured at 600 nm using a photometer from FoodALYT. Cell lysis was performed using a SONOPULS ultrasonic homogenizer from Bandelin. IMAC was performed with column material from Macherey-Nagel GmbH & Co. KG containing Ni ions, such as Ni-NTA. Buffer exchange was performed using a column from GE Healthcare AB (V= 8.3 mL). A Merck KGaA filter unit with an exclusion limit of 30000 Da was used to constrict protein solutions. The extinction coefficient for protein concentration measurement was determined using ProtParam (ExPASy). UV/Vis spectroscopy was performed at 280 nm using a DeNovix spectrophotometer (type: DS-11+). GC-MS analyses for in vitro biotransformations were performed using an Agilent 5977B GC/MSD with 7890B GC system and an Optima 5HT - 0.25 µm, carrier gas: He, column volume: 30 m x 250 µm x 0.25 µm, injection volume: 1 µL. Retention indices (RI) were determined in comparison to n-alkanes (C7 to C30).

Composition of buffer solutions und culture media

In order to set pH value for the buffer solutions, aqueous NaOH and HCl solutions were used.

Lysis buffer:	Tris·HCl (40 mM), NaCl (100 mM)
Ni-NTA buffer (x M):	Tris·HCl (40 mM), NaCl (100 mM), Imidazol (x M)
HEPES buffer:	HEPES (50 mM), DTT (5 mM), pH =7.5
LB media:	0.50% (w/v) yeast extract (Duchefa Biochemie)
	1.00% (w/v) trypton (Duchefa Biochemie)
	0.50% (w/v) NaCl (Roth or VWR)
2-TY media:	1.00% (w/v) yeast extract (Duchefa Biochemie)
	1.60% (w/v) trypton (Duchefa Biochemie)
	0.50% (w/v) NaCl (Roth or VWR)

Information on and characterization of BcBot2

The gene for BcBot2 used in this work was synthesized by GENEWIZ LLC as a construct with pUC57 and are optimized for protein expression in *E.coli*. The gene sequence can be used from a gene bank for the accession number Q6WP50.[1]

Information on and characterization of GCoA

The gene for GCoA used in this work was synthesized by GENEWIZ LLC as a construct with pUC57 and are optimized for protein expression in *E.coli*. The gene sequence can be used from a gene bank for the accession number B1W019.[2]

1.2 Chemical synthesis

***tert*-Butyl(hex-5-yn-1-yloxy)diphenylsilane S1[3,4]**

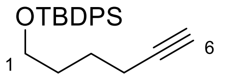

S1
$C_{22}H_{28}OSi$
336.55

Hex-5-yn-1-ol (**10**) (168 mg, 1.71 mmol, 1.00 eq.) was dissolved in CH_2Cl_2 (3 mL) and cooled to 0 °C. Imidazole (233 mg, 3.42 mmol, 2.00 eq.) was added in two portions and the solution has been stirred for 15 min at 0 °C. After the addition of TBDPSCl (0.54 mL, 571 mg, 2.08 mmol, 1.21 eq.) the ice bath was not renewed and the reaction mixture was allowed to slowly warm up to rt for 3.5 h. The reaction was terminated by the addition of water and the phases were separated. The aqueous phase was extracted with EtOAc. The combined organic phases were washed with brine, dried over $MgSO_4 \cdot H_2O$, filtered and the solvent was removed *in vacuo*. The crude product was purified by column chromatography (PE : EtOAc= 9:1) and alkyne **S1** (547 mg, 1.63 mmol; 95%) was obtained as a colorless oil.[4]

R_f = 0.64 (PE : EtOAc= 10:1); ^{1}H NMR (400 MHz, $CDCl_3$): δ = 7.69 – 7.65 (m, 4H, H_{Ar}), 7.45 – 7.36 (m, 6H, H_{Ar}), 3.68 (t, J = 6.0 Hz, 2H, H_1), 2.19 (td, J = 6.8 Hz, 2.7 Hz, 2H, H_4), 1.94 (t, J = 2.7 Hz, 1H, H_6), 1.71 – 1.60 (m, 4H, H_2, H_3), 1.05 (s, 9H, $H_{tert\text{-Bu}}$) ppm; ^{13}C NMR (101 MHz, $CDCl_3$): δ = 135.7 (C_{Ar}), 134.1 (C_{Ar}), 129.7 (C_{Ar}), 127.8 (C_{Ar}), 84.7 (C_5), 68.4 (C_6), 63.5 (C_1), 31.7 ($C_{2/3}$), 27.0 ($C_{tert\text{-Bu}}$),

25.1 ($C_{2/3}$), 19.4 ($C_{tert\text{-}Bu}$), 18.3 (C_4) ppm; GC-MS [EI, 13.055 min] *m/z* (%): 199.1 (100), 200 (25), 201 (22), 279.1 (17, *t*-Bu fragmentation).

(*E*)-7-((*tert*-Butyldiphenylsilyl)oxy)-3-methylhept-2-en-1-ol 11[4,5]

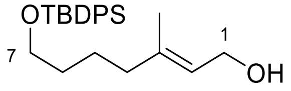

11
$C_{24}H_{34}O_2Si$
382.62

Alkyne **S1** (2.50 g, 7.43 mmol, 1.00 eq.) was dissolved in CH_2Cl_2 (5 mL), cooled to 0 °C and $AlMe_3$ (1 M in heptane, 2.6 mL, 2.60 mmol, 0.35 eq.) was added to the solution which then was stirred for 30 min. Meanwhile, $ZrCp_2Cl_2$ (883 mg, 3.02 mmol, 0.41 eq.) was dissolved in CH_2Cl_2 (25 mL) at 0 °C. $AlMe_3$ (1 M in heptane, 21.0 mL, 21.0 mmol, 2.83 eq.) and H_2O (0.14 mL, 140 mg, 7.77 mmol, 1.05 eq.) were added and the solution was stirred for 15 min at 0 °C before the alkyne-$AlMe_3$-solution is being added. The reaction mixture was stirred at rt for 12 h, cooled to 0 °C and a suspension of paraformaldehyde (1.14 g, 37.5 mmol, 5.05 eq.) in CH_2Cl_2 (10 mL + 5 mL) was added. After stirring for 1 h at 0 °C, the reaction mixture was warmed up to rt and stirring was continued for additional 1.5 h. The reaction was terminated by the addition of a sat. aq. Rochelle salt solution. After stirring at rt and dilution with EtOAc, the phases were separated and the aqueous phase was extracted with EtOAc (3x). The combined organic phases were washed with brine, dried over $MgSO_4 \cdot H_2O$, filtered and the solvent was removed *in vacuo*. The crude product was purified by column chromatography (PE : EtOAc= 3:1) and alcohol **11** (2.34 g, 6.11 mmol; 82%) was obtained as a colorless oil with small amounts of EtOAc.[4]

R_f = 0.24 (*n*-pentane : Et_2O= 3:1); ^{1}H NMR (400 MHz, $CDCl_3$): δ = 7.68 – 7.65 (m, 4H, H_{Ar}), 7.45 – 7.36 (m, 6H, H_{Ar}), 5.38 (tq, *J* = 7.0 Hz, 1.3 Hz, 1H, H_2), 4.14 (dd, *J* = 5.9 Hz, 5.9 Hz, 2H, H_1), 3.66 (t, *J* = 6.0 Hz, 2H, H_7), 2.00 (t, *J* = 6.9 Hz, 2H, H_4), 1.56 (s, 3H, H_8), 1.58 – 1.45 (m, 4H, H_5, H_6), 1.07 (t, *J* = 5.3 Hz, 1H, H_{OH}, superposition with 1.05), 1.05 (s, 9H, $H_{tert\text{-}Bu}$, superposition with 1.07) ppm; ^{13}C NMR (101 MHz, $CDCl_3$): δ = 140.1 (C_3), 135.7 (C_{Ar}), 134.2 (C_{Ar}), 129.7 (C_{Ar}), 127.7 (C_{Ar}), 123.5 (C_2), 63.9 (C_7), 59.6 (C_1), 39.3 (C_4), 32.2 ($C_{5/6}$), 27.0 ($C_{tert\text{-}Bu}$), 23.9 ($C_{5/6}$), 19.4 ($C_{tert\text{-}Bu}$), 16.2 (C_8) ppm; HRMS [ESI-MS]: *m/z* calcd for $C_{24}H_{34}O_2SiNa$ $[M+Na]^+$: 405.2226, found: 405.2220.

(*E*)-*tert*-Butyl((5-methyl-7-((tetrahydro-2*H*-pyran-2-yl)oxy)hept-5-en-1-yl)oxy)diphenylsilane S2

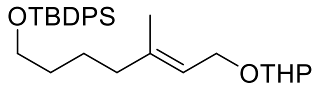

S2
$C_{29}H_{42}O_3Si$
466.74

Alcohol **11** (2.32 g, 6.07 mmol, 1.00 eq.) was dissolved in CH_2Cl_2 (20 mL) and cooled to 0 °C. PPTS (154 mg, 0.61 mmol, 1.01 eq.) and DHP (1.00 mL, 0.92 g, 10.9 mmol, 1.80 eq.) were added to the solution and stirring was continued at 0 °C for 30 min, before warming up to rt over a period of additional 5 h. The reaction was terminated by adding a sat. aq. $NaHCO_3$-solution, diluted with Et_2O and the phases were separated. The combined organic phases were extracted with Et_2O (3x), dried over $MgSO_4 \cdot H_2O$, filtered and the solvent was removed *in vacuo*. The crude product was purified by column chromatography (PE : EtOAc= 17:1) and THP ether **S2** (2.51 g, 5.38 mmol; 89%) was obtained as a colorless oil.

R_f = 0.40 (*n*-pentane : Et_2O= 10:1); ^{1}H NMR (400 MHz, $CDCl_3$): δ = 7.68 – 7.65 (m, 4H, H_{Ar}), 7.44 – 7.36 (m, 6H, H_{Ar}), 5.34 (t, *J* = 6.9 Hz, 1H, H_6), 4.63 (dd, *J* = 4.5 Hz, 3.3 Hz, 1H, H_{THP}), 4.23 (dd, *J* = 12.0 Hz, 6.4 Hz, 1H, H_7), 4.03 (dd, *J* = 11.9 Hz, 7.4 Hz, 1H, H_7), 3.92 – 3.87 (m, 1H, H_{THP}), 3.66 (t, *J* = 6.0 Hz, 2H, H_1), 3.54 – 3.47 (m, 1H, H_{THP}), 2.01 (t, *J* = 7.0 Hz, 2H, H_4), 1.88 – 1.80 (m, 1H, H_{THP}), 1.75 – 1.68 (m, 1H, H_{THP}), 1.65 (s, 3H, H_8), 1.63 – 1.48 (m, 8H, $H_{THP,}$ H_2, H_3), 1.05 (9H, s, $H_{tert\text{-}Bu}$) ppm; ^{13}C NMR (101 MHz, $CDCl_3$): δ = 140.5 (C_5), 135.7 (C_{Ar}), 134.3 (C_{Ar}), 129.6 (C_{Ar}), 127.7 (C_{Ar}), 120.8 (C_6), 98.0 (C_{THP}), 63.9 (C_1), 63.8 (C_7), 62.5 (C_{THP}), 39.4 (C_4), 32.3 ($C_{2/3}$), 30.9 (C_{THP}), 27.0 ($C_{tert\text{-}Bu}$), 25.7

(C_{THP}), 23.9 ($C_{2/3}$), 19.8 (C_{THP}), 19.4 ($C_{tert\text{-}Bu}$), 16.4 (C_8) ppm; HRMS [ESI-MS]: *m/z* calcd for $C_{29}H_{42}O_3SiNa$ $[M+Na]^+$: 489.2801, found: 489.2788.

(*E*)-5-Methyl-7-((tetrahydro-2*H*-pyran-2-yl)oxy)hept-5-en-1-ol S3

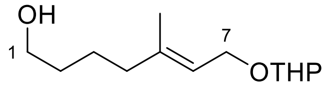

S3
$C_{13}H_{24}O_3$
228.33

THP Ether **S2** (2.49 g, 5.34 mmol, 1.00 eq.) was dissolved in THF (20 mL) and cooled to 0 °C. TBAF (1 M in THF, 8 mL, 8.00 mmol, 1.50 eq.) was added and the reaction mixture was warmed to rt after stirring for 15 min at 0 °C. The solution was stirred for 2.5 h before the reaction was terminated by the addition of water, diluted with EtOAc and the phases were separated. The aqueous phase was extracted with EtOAc (3x) and the combined organic phases were washed with brine, dried over $MgSO_4 \cdot H_2O$, filtered and the solvent was removed *in vacuo*. The crude product was purified by column chromatography (PE : EtOAc= 1:1) and alcohol **S3** (1.02 g, 4.48 mmol; 84%) was obtained as a yellow oil.

R_f = 0.37 (*n*-pentane : Et_2O= 1:1); ^1H NMR (400 MHz, $CDCl_3$): δ = 5.36 (t, J = 6.9 Hz, 1H, H_6), 4.62 (dd, J = 4.2 Hz, 2.9 Hz, 1H, H_{THP}), 4.24 (dd, J = 11.9 Hz, 6.4 Hz, 1H, H_7), 4.02 (dd, J = 11.8 Hz, 7.3 Hz, 1H, H_7), 3.92 – 3.86 (m, 1H, H_{THP}), 3.65 (t, J = 6.1 Hz, 2H, H_1), 3.54 – 3.48 (m, 1H, H_{THP}), 2.06 (t, J = 7.1 Hz, 2H, H_4), 1.86 – 1.78 (m, 1H, H_{THP}), 1.75 – 1.68 (m, 1H, H_{THP}), 1.67 (s, 3H, H_8), 1.62 – 1.46 (m, 8H, H_2, H_3, H_{THP}) ppm; ^{13}C NMR (101 MHz, $CDCl_3$): δ = 140.1 (C_5), 121.0 (C_6), 98.0 (C_{THP}), 63.8 (C_7), 63.0 (C_1), 62.5 (C_{THP}), 39.4 (C_4), 32.5 ($C_{2/3}$), 30.9 (C_{THP}), 25.6 (C_{THP}), 23.9 ($C_{2/3}$), 19.8 (C_{THP}), 16.4 (C_8) ppm; HRMS [ESI-MS]: *m/z* calcd for $C_{13}H_{24}O_3Na$ $[M+Na]^+$: 251.1623, found: 251.1624.

(*E*)-5-Methyl-7-((tetrahydro-2*H*-pyran-2-yl)oxy)hept-5-enal 12

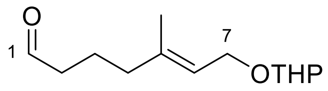

12
$C_{13}H_{22}O_3$
226.32

CH_2Cl_2 (30 mL) was cooled to −78 °C, oxalylchloride (1.90 mL, 2.85 g, 22.5 mmol, 2.53 eq.) and DMSO (1.60 mL, 1.76 g, 22.5 mmol, 2.54 eq.) were added and after 10 min alcohol **S3** (2.03 g, 8.87 mmol, 1.00 eq.) dissolved in CH_2Cl_2 (5 mL + 10 mL) was added to the mixture. After additional 10 min Et_3N (6.5 mL, 4.72 g, 46.6 mmol, 5.23 eq.) was added and the reaction mixture was stirred for additional 5 min at −78 °C before being warmed up to rt. After 30 min the reaction was terminated by the addition of water and EtOAc. The phases were separated, the aqueous phase was extracted with EtOAc (3x) and the combined organic phases were washed with brine, dried over $MgSO_4 \cdot H_2O$, filtered and the solvent was removed *in vacuo*. The crude product was purified by column chromatography (PE : EtOAc= 3:1) and aldehyde **12** (1.83 g, 8.09 mmol, 91%) was obtained as a pale yellow oil.

R_f = 0.67 (*n*-pentane : Et_2O= 1:1); ^1H NMR (400 MHz, $CDCl_3$): δ = 9.79 (t, J = 1.6 Hz, 1H, H_1), 5.42 – 5.37 (m, 1H, H_6), 4.64 (dd, J = 4.2 Hz, 2.9 Hz, 1H, H_{THP}), 4.26 (dd, J = 12.0 Hz, 6.4 Hz, 1H, H_7), 4.04 (dd, J = 12.0 Hz, 7.2 Hz, 1H, H_7), 3.93 – 3.88 (m, 1H, H_{THP}), 3.56 – 3.50 (m, 1H, H_{THP}), 2.44 (td, J = 7.3 Hz, 1.6 Hz, 2H, H_2), 2.09 (t, J = 7.5 Hz, 2H, H_4), 1.92 – 1.71 (m, 4H, H_{THP}, H_3), 1.69 (s, 3H, H_8), 1.65 – 1.52 (m, 4H, H_{THP}) ppm; ^{13}C NMR (101 MHz, $CDCl_3$): δ = 202.6 (C_1), 139.0 (C_5), 121.9 (C_6), 98.1 (C_{THP}), 63.7 (C_7), 62.5 (C_{THP}), 43.4 (C_2), 38.9 (C_4), 30.9 (C_{THP}) 25.6 (C_{THP}), 20.1 (C_3), 19.8 (C_{THP}), 16.6 (C_8) ppm; HRMS [ESI-MS]: *m/z* calcd for $C_{13}H_{22}O_3Na$ $[M+Na]^+$: 249.1467, found: 249.1464.

(*E*)-2,10-dimethyl-12-((tetrahydro-2*H*-pyran-2-yl)oxy)dodeca-2,10-dien-6-ol S4

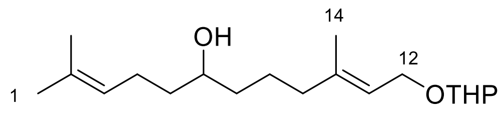

S4
$C_{19}H_{34}O_3$
310.48

Bromide **15** (101 mg, 0.62 mmol, 1.41 eq.) was dissolved in Et_2O (6 mL) at −78 °C and *t*-BuLi (1.71 M in pentane, 0.8 mL, 1.37 mmol, 3.11 eq.) was slowly added and the reaction mixture was stirred for 1 h at this temperature. Aldehyde **12** (99 mg, 0.44 mmol, 1.00 eq.) was dissolved in Et_2O (1 mL + 2 mL) and added to the reaction mixture, that was warmed up to rt after 5 min. After 2 h the solvent was removed and the residue was taken up with Et_2O and H_2O. The phases were separated and the aqueous phase was extracted with Et_2O (3x). The combined organic phases were dried over $MgSO_4 \cdot H_2O$, filtered and the solvent was removed *in vauco*. The crude product was purified by column chromatography (*n*-pentane : Et_2O= 1:1) and alcohol **S4** (83 mg, 0.27 mmol, 61%) was obtained as a pale yellow oil.

R_f = 0.59 (PE : EtOAc= 2:1); ^{1}H NMR (400 MHz, $CDCl_3$): δ = 5.36 (t, J = 6.9 Hz, 1H, H_{11}), 5.13 (t, J = 7.2 Hz, 1H, H_3), 4.62 (dd, J = 4.1 Hz, 2.8 Hz, 1H, H_{THP}), 4.23 (dd, J = 12.0 Hz, 6.4 Hz, 1H, H_{12}), 4.02 (dd, J = 11.9 Hz, 7.3 Hz, 1H, H_{12}), 3.92 – 3.86 (m, 1H, H_{THP}), 3.64 – 3.58 (m, 1H, H_6), 3.54 – 3.48 (m, 1H, H_{THP}), 2.17 – 1.97 (m, 4H, H_4, H_9), 1.88 – 1.78 (m 1H, H_{THP}), 1.75 – 1.67 (m, 7H, H_{THP}, $H_{1/13}$, H_{14}), 1.63 (s, 3H, $H_{1/13}$), 1.61 – 1.36 (m, 10H, H_{THP}, H_5, H_7, H_8) ppm; ^{13}C NMR (101 MHz, $CDCl_3$): δ = 140.3 (C_{10}), 132.3 (C_2), 124.3 (C_3), 120.9 (C_{11}), 98.0 (C_{THP}), 71.8 (C_6), 63.8 (C_{12}), 62.5 (C_{THP}), 39.7 (C_9), 37.5 ($C_{THP/5/7/8}$), 37.2 ($C_{THP/5/7/8}$), 30.9 ($C_{THP/5/7/8}$), 25.9 ($C_{1/13}$), 25.7 ($C_{THP/5/7/8}$), 24.5 (C_4), 23.8 ($C_{THP/5/7/8}$), 19.8 ($C_{THP/5/7/8}$), 17.8 ($C_{1/13}$), 16.4 (C_{14}) ppm; HRMS [ESI-MS]: *m/z* calcd for $C_{19}H_{34}O_3Na$ $[M+Na]^+$: 333.2406, found: 333.2401.

(*E*)-2,10-Dimethyl-12-((tetrahydro-2*H*-pyran-2-yl)oxy)dodeca-2,10-dien-6-one 13

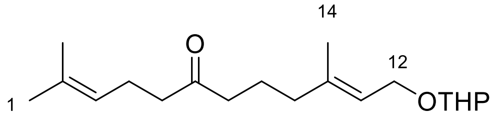

13
$C_{19}H_{32}O_3$
308.46

At −78 °C oxalyl chloride (0.07 mL, 105 mg, 0.83 mmol, 2.43 eq.) was dissolved in CH_2Cl_2 (5 mL) and DMSO (0.06 mL, 66 mg, 0.84 mmol, 2.46 eq.) was added. After stirring for 10 min at −78 °C alcohol **S4** (106 mg, 0.34 mmol, 1.00 eq.) dissolved in CH_2Cl_2 (1 mL + 2 mL) was added to the reaction mixture. After another 30 min Et_3N (0.24 mL, 174 mg, 1.72 mmol, 5.05 eq.) was added and the reaction mixture was stirred for 10 min at −78 °C and then 35 min at rt. The reaction was terminated by the addition of water and diluted with EtOAc and brine. The phases were separated and the aqueous phase was extracted with EtOAc (3x). The combined organic phases were dried over $MgSO_4 \cdot H_2O$, filtered and the solvent was removed *in vacuo*. The crude product was purified by column chromatography (PE : EtOAc= 8:1) and ketone **13** (87 mg, 0.28 mmol, 83%) was obtained as a colorless-pale yellow oil.

R_f = 0.40 (PE : EtOAc= 7:1); ^{1}H NMR (400 MHz, $CDCl_3$): δ = 5.35 (t, J = 6.8 Hz, 1H, H_{11}), 5.05 (t, J = 7.1 Hz, 1H, H_3), 4.61 (dd, J = 4.2 Hz, 2.9 Hz, 1H, H_{THP}), 4.23 (dd, J = 12.1 Hz, 6.4 Hz, 1H, H_{12}), 4.01 (dd, J = 12.1 Hz, 7.3 Hz, 1H, H_{12}), 3.91 (m, 1H, H_{THP}), 3.54 – 3.48 (m, 1H, H_{THP}), 2.41 (t, J = 7.5Hz, 2H, H_5), 2.37 (t, J = 7.4 Hz, 2H, H_7), 2.24 (dt, J = 7.2 Hz, 7.2 Hz, 2H, H_4), 2.02 (t, J = 7.5 Hz, 2H, H_9), 1.88 – 1.79 (m, 1H, H_{THP}), 1.75 – 1.69 (m, 3H, H_{THP}, H_8), 1.68 – 1.66 (m, 3H, $H_{1/13}$), 1.66 (s, 3H, H_{14}), 1.61 (s, 3H, $H_{1/13}$), 1.60 – 1.48 (m, 4H, H_{THP}) ppm; ^{13}C NMR (101 MHz, $CDCl_3$): δ = 211.0 (C_6), 139.5 (C_{10}), 132.8 (C_2), 123.0 (C_3), 121.5 (C_{11}), 98.1 (C_{THP}), 63.8 (C_{12}), 62.5 (C_{THP}), 43.0 (C_5), 42.3 (C_7), 39.1

(C_9), 30.9 (C_{THP}), 25.8 ($C_{1/13}$), 25.6 (C_{THP}), 22.7 (C_4), 21.7 (C_8), 19.8 (C_{THP}), 17.8 ($C_{1/13}$), 16.3 (C_{14}) ppm; HRMS [ESI-MS]: *m/z* calcd for $C_{19}H_{32}O_3Na$ $[M+Na]^+$: 331.2249, found: 331.2238.

(*E*)-2-((3,11-Dimethyl-7-methylenedodeca-2,10-dien-1-yl)oxy)tetrahydro-2*H*-pyran S5

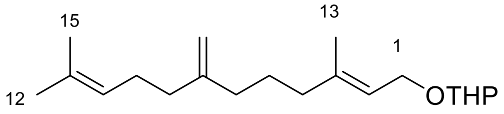

S5
$C_{20}H_{34}O_2$
306.49

Methyltriphenylphosphonium bromide (2.45 g, 6.85 mmol, 4.54 eq.) was suspended in THF (8 mL) at 0 °C. *n*-BuLi (1.6 M in hexane, 3.8 mL, 6.08 mmol, 4.03 eq.) was added and the reaction mixture was stirred for 10 min at 0 °C and 1.5 h at rt resulting in a dark orange suspension. Ketone **13** (465 mg, 1.51 mmol, 1.00 eq.) was dissolved in THF (1.5 mL + 1.5 mL) and added to the reaction mixture and stirring was continued for approx. 43 h at rt. Then, water and Et_2O were added. The phases were separated, the aqueous phase was extracted with Et_2O (3x) and the combined organic phases were washed with brine, dried over $MgSO_4 \cdot H_2O$ and the solvent was removed *in vacuo*. The crude product was purified by column chromatography (PE : EtOAc= 20:1 → 10:1) and alkene **S5** (375 mg, 1.22 mmol, 81%) was obtained as a colorless oil. Additionally, ketone **13** (48 mg, 0.16 mmol, 10%) was partially reisolated resulting in a yield of 91% brsm.

R_f = 0.66 (PE : EtOAc= 7:1); ^{1}H NMR (400 MHz, $CDCl_3$): δ = 5.36 (t, J = 6.9 Hz, 1H, H_2), 5.11 (t, J = 6.8 Hz, 1H, H_{10}), 4.72 (s, 2H, H_{14}), 4.63 (dd, J = 4.5 Hz, 2.9 Hz, 1H, H_{THP}), 4.24 (dd, J = 11.9 Hz, 6.4 Hz, 1H, H_1), 4.03 (dd, J = 11.9 Hz, 7.4 Hz, 1H, H_1), 3.92 – 3.87 (m, 1H, H_{THP}), 3.54 – 3.48 (m, 1H, H_{THP}), 2.13 – 2.08 (m, 2H, H_9), 2.04 – 1.98 (m, 6H, H_4, H_6, H_8), 1.88 – 1.80 (m, 1H, H_{THP}), 1.76 – 1.70 (m, 1H, H_{THP}), 1.69 (s, 3H, $H_{12/15}$), 1.67 (s, 3H, H_{13}), 1.63 – 1.49 (m, 9H, $H_{12/15}$, H_5, H_{THP}) ppm; ^{13}C NMR (101 MHz, $CDCl_3$): δ = 149.7 (C_7), 140.4 (C_3), 131.7 (C_{11}), 124.3 (C_{10}), 120.8 (C_2), 109.0 (C_{14}), 98.0 (C_{THP}), 63.8 (C_1), 62.5 (C_{THP}), 39.5 (C_4), 36.2 (C_8), 35.9 (C_6), 30.9 (C_{THP}), 26.6 (C_9), 25.9 (C_5), 25.8 ($C_{12/15}$), 25.7 (C_{THP}), 19.8 (C_{THP}), 17.9 ($C_{12/15}$), 16.5 (C_{13}) ppm; HRMS [ESI-MS]: *m/z* calcd for $C_{20}H_{34}O_2Na$ $[M+Na]^+$: 329.2457, found: 329.2453.

(*E*)-3,11-Dimethyl-7-methylenedodeca-2,10-dien-1-ol 14[6]

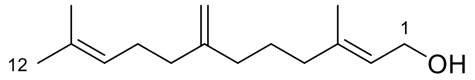

14
$C_{15}H_{26}O$
222.37

Alkene **S5** (374 mg, 1.22 mmol, 1.00 eq.) was dissolved in dry EtOH and heated to 50 °C. PPTS (92 mg, 0.37 mmol, 0.30 eq.) was added and the reaction mixture was stirred for 2.5 h at 50 °C. Water and Et_2O were added, the phases were separated and the aqueous phase was extracted with Et_2O (5x). The combined organic phases were dried over $MgSO_4 \cdot H_2O$, filtered and the solvent was removed *in vacuo*. The crude product was purified by column chromatography (PE : EtOAc= 8:1 → 4:1) and alcohol **14** (253 mg, 1.14 mmol, 93%) was obtained as a pale orange oil.[6]

R_f = 0.21 (PE : EtOAc= 5:1); ^{1}H NMR (400 MHz, $CDCl_3$): δ = 5.42 (t, J = 7.0 Hz, 1H, H_2), 5.11 (t, J = 6.8 Hz, 1H, H_{10}), 4.73 (s, 2H, H_{14}), 4.16 (dd, J = 5.5 Hz, 5.5 Hz, 2H, H_1), 2.14 – 2.08 (m, 2H, H_9), 2.04 – 1.98 (m, 6H, H_4, H_6, H_8), 1.69 (s, 3H, $H_{12/15}$), 1.68 (s, 3H, H_{13}), 1.61 (s, 3H, $H_{12/15}$), 1.60 – 1.52 (m, 2H, H_5) ppm; ^{13}C NMR (101 MHz, $CDCl_3$): δ = 149.6 (C_7), 140.0 (C_3), 131.7 (C_{11}), 124.3 (C_{10}), 123.5 (C_2), 109.1 (C_{14}), 59.6 (C_1), 39.4 (C_4), 36.2 ($C_{6/8}$), 35.9 ($C_{6/8}$), 26.6 (C_9), 25.9 (C_5), 25.8 ($C_{12/15}$), 17.9 ($C_{12/15}$), 16.3 (C_{13}) ppm; HRMS [GC-MS, EI]: *m/z* calcd for $C_{15}H_{24}$ $[M-H_2O]^+$: 204.1878, found: 204.1870.

FPP-derivative 9[6]

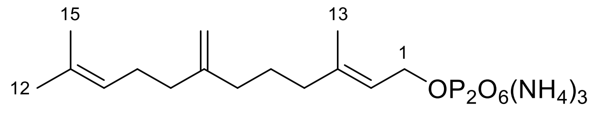

9
$C_{15}H_{37}N_3O_7P_2$
433.42

DMS (0.07 mL, 59 mg, 0.95 mmol, 2.17 eq.) was added to a suspension of NCS (127 mg, 0.95 mmol, 2.16 eq.) in CH_2Cl_2 (10 mL) at 0 °C. After 10 min alcohol **14** (97 mg, 0.44 mmol, 1.00 eq.) was dissolved in CH_2Cl_2 (3 mL + 2 mL) and added to the reaction mixture. After 1 h at 0 °C brine and Et_2O were added and the phases were separated. The aqueous phase was extracted with Et_2O (3x) and the combined organic phases were dried over $MgSO_4{\cdot}H_2O$, filtered and the solvent was carefully removed *in vacuo*. $((n\text{-Bu})_4N)_3O_7P_2H$ was dissolved in MeCN (5 mL) and the crude chloride was dissolved in MeCN (3 mL + 3 mL) and added to the solution. The reaction mixture was stirred at rt o/n. The solvent was removed *in vacuo* and the residue was used for the ion exchange purification (see general information) resulting in FPP-derivative **9** (105 mg, 0.24 mmol, 55%) which was found to be a white/yellow fluffy solid. The solvent signal (D_2O) in the ^{1}H NMR spectrum was supressed to enhance the product signal visibility, H_{14} is superposed by the solvent signal and can only be seen in 2D NMR spectra.[6]

^{1}H NMR (400 MHz, D_2O): δ = 5.48 (t, J = 7.2 Hz, 1H, H_2), 5.22 (t, J = 6.8 Hz, 1H, H_{10}), 4.82 (2H, H_{14}), 4.49 (dd, J = 6.5 Hz, 6.5 Hz, 2H, H_1), 2.19 – 2.03 (m, 8H, H_4, H_6, H_8, H_9), 1.73 (s, 3H, H_{13}), 1.70 (s, 3H, $H_{12/15}$), 1.64 (s, 3H, $H_{12/15}$), 1.62 – 1.56 (m, 2H, H_5) ppm; ^{13}C NMR (101 MHz, D_2O): δ = 151.5 (C_7), 143.0 (C_3), 133.6 (C_{11}), 124.4 (C_{10}), 119.8 (d, J = 8.7 Hz, C_2), 108.7 (C_{14}), 62.5 (d, J = 5.2 Hz, C_1), 38.5 (C_4), 35.2 ($C_{6/8}$), 34.9 ($C_{6/8}$), 25.6 (C_9), 25.1 (C_5), 24.8 ($C_{12/15}$), 16.9 ($C_{12/15}$), 15.5 (C_{13}) ppm; ^{31}P NMR (162 MHz, D_2O): δ = −7.1 (d, J = 20.3 Hz, 1P), −10.1 (d, J = 21.3 Hz, 1P).

1.3 Microbiological methods and biotransformations

Heterologous proteinexpression and cell lysis *via* ultrasound

In order to cultivate the *E.coli* BL21 (DE3) cells, carrying the required plasmids, a seed culture (50 µL) was incubated with Kanamycin (50 mg/mL, 3 µL) in LB-media (3 mL) for 4.5 h at 37 °C and 200 rpm. Alternatively a seed culture (5 µL) can be incubated with Kanamycin (50 mg/mL, 5 µL) in LB-Media (5 mL) at 37 °C and 180 rpm o/n. From this pre-culture (1 mL) a main culture was created by incubation with Kanamycin (50 mg/mL) in 2-TY media (50 mL) at 37 °C and 200 rpm until the culture reached an OD_{600} value of 0.4 to 0.8. To initiate the protein overexpression IPTG (1 M, 25 µL) was added to the culture that was stirred at 16 °C and 180 rpm for approximately 22 h. After centrifugation, the cell pellets were stored at −20 °C or used immediately for cell lysis. Cells were resuspended in lysis buffer (20 mL) at 0 °C and lysed by ultrasonication (10 min, 45% amplitude, 4 s ultrasound to 6 s pause). The resulting solution was centrifuged (4 °C, 20 min, 10000 g) to give the crude enzyme solution.

Immobilized metal-affinity chromatography

For conditioning, the column was rinsed with water (10x the column volume) and lysis buffer (5x column volume). The lysate was loaded onto the column (2x) and eluted with Ni-NTA buffers (5 mL each) with increasing imidazol concentrations (25 mM, 50 mM, 100 mM, 250 mM, 500 mM). During this time the solutions were cooled at 0 °C. The fractions were analyzed using a Brentford assay and those fractions containing protein were united and concentrated by centrifugation (20 min, 4 °C, 4500 rpm).

Buffer exchange

To perform the buffer exchange the column was rinsed with water (10x column volume) and HEPES buffer (5x column volume). The protein solutions were loaded onto the column and eluted with HEPES buffer (5 mL). After centrifugation (4 °C, 4500 rpm) the solutions can be used or stored as a mixture of water and glycerol (1/1) between −70 °C and −80 °C.

Concentration measurement

Concentrations were determined by measuring the absorption ($\lambda = 280$ nm) of the purified protein solutions, using the extinction coefficient for reduced cysteine side chains.

***In-vitro* biotransformation in an analytical scale**

In order to find STCs that are able to accept FPP-derivative **9** as a substrate we tested nine different enzymes, namely presilphiperfolan-8-β-ol synthase (BcBot2), pentalenene synthase (PenA), trichodiene synthase (Tri5), epi-isozizaene synthase (Cyc1), vetispiradiene synthase (Hvs1), caryolan-1-ol-synthase (GcoA), viridiflorene synthase (Tps32), Δ6-protoilludene synthases (Omp7), cubebol synthase (Cop4). Since GCoA and BcBot2 turned out to be most susceptible towards **9** with good ratios in favor of the main product versus the minor by-products. The protocol for these enzymes will be described in detail below. Screening for new biotransformation products was performed on a reaction scale of 500 µL containing the corresponding enzyme (0.05 mg), the FPP-derivative **9** (1.50 µL, 50 mM) and a $MgCl_2$ solution (1.25 µL, 2 M). In parallel also negative (without derivative or without enzyme) and positive controls (reaction of FPP (**1**) with different enzymes) under analogous conditions were carried out. All reactions were carried out in HEPES buffer. The reactions were incubated for 70 min at 37 °C and 100 rpm (30 °C, 0 rpm for FPP). In order to extract the products n-hexane (100 µL) was added and the phases were separated by centrifugation (3000 rpm, 6 min, 4 °C). The hexane extract was used for GC-MS analysis.

In Table S1, the different products and their relative abundance are listed that are formed by BcBOT2 when exposed to FPP derivative **9**.

Table S1. Retention indices (RI) and the approximate relative abundance to **16** (highlighted) measured on $DB5_{HT}$ for the analytical scale biotransformation extract of BcBOT2 and 7-methylene farnesylpyrophosphate (**9**).

t_R [min] (FID)	RI_{DB5HT}	rel. abund. in [%] (TIC)	t_R [min] (FID)	RI_{DB5HT}	rel. abund. in [%] (TIC)
8.48	1380	1	9.47	1546	2
8.57	1393	12	9.60	1558	3
8.73	1418	4	9.94	1613	3
8.94	1450	4	10.16	1653	100

Biotransformation for product isolation with BcBot2

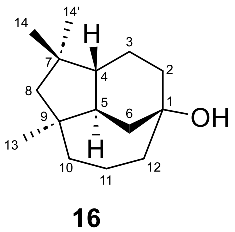

16

In order to obtain sufficient amounts of the biotransformation product detected in the analytical studies for the purpose of structural elucidation, the reaction was repeated on a 60 mL scale and the total volume was divided into two parallel reactions. These were composed of the following components: Tween® 20 (5 µL each) and diphosphatase (0.5 µL each) in HEPES buffer (26.8 mL). The BcBOT2 solution (314 µL, 4.78 g/mol), derivative solution (42.4 mg in 5.22 mL NH_4HCO_3 (0.05 M), 300 µL) and finally $MgCl_2$ (2 M, 150 µL) were added and the reaction was shaken (100 rpm) at 37 °C. Every 30 minutes a

solution (250 µL) of derivative **9** was added up to a total volume of 2.4 mL per batch. After 2 h the BcBOT2-containing solution (314 µL each) was added the reaction mixture was shaken (100 rpm) at 37 °C overnight. *n*-Pentane was added and the individual batches were combined. The phases were separated and the aqueous phase was extracted with *n*-pentane (3x) using ultrasound to accelerate separation. The combined organic phases were washed with brine, dried over $MgSO_4 \cdot H_2O$ and the solvent was removed *in vacuo*. The crude product was purified by column chromatography (*n*-pentane 100% → *n*-pentane : MTBE = 9:1 → 5:1 → 2:1 → MTBE 100%) to yield terpenoid **16** (1 mg) as a colorless oil.

$R_f = 0.18$ (*n*-pentane : MTBE= 5:1); ^{1}H NMR (600 MHz, C_6D_6): $\delta = 2.00$ (dd, $J = 15.4$ Hz, 7.0 Hz, 1H, H_{12}), 1.96 (d, $J = 12.2$ Hz, 1H, H_6), 1.82 – 1.74 (m, 2H, H_2, H_{12}), 1.70 – 1.63 (m, 3H, H_2, H_{11}, H_4), 1.56 – 1.48 (m, 2H, H_3, H_{10}), 1.42 – 1.24 (m, 6H, H_5, H_6, H_8, H_{10}, H_{11}), 1.22 – 1.15 (m, 1H, H_3), 1.04 (s, 3H, H_{14}), 0.95 (s, 3H, H_{13}), 0.77 (s, 3H, $H_{14'}$) ppm; ^{13}C NMR (151 MHz, C_6D_6): $\delta = 73.7$ (C_1), 58.3 (C_8), 49.2 (C_4), 48.3 (C_{12}), 47.5 (C_5), 42.3 (C_2), 42.0 (C_{10}), 39.5 (C_9), 35.8 (C_7), 33.8 (C_6), 30.8 (C_{14}), 26.6 (C_{13}), 25.0 ($C_{14'}$), 22.4 (C_{11}), 20.3 (C_3) ppm; HRMS [GC-MS, CI]: *m/z* calcd for $C_{15}H_{26}O$ $[M]^+$: 222.1984, found: 222.1994; $R_I = 1678$.

Biotransformation for product isolation with GCoA

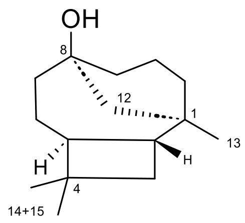

17

In order to obtain sufficient amounts of the biotransformation product detected in the analytical studies for the purpose of structural elucidation, the reaction was repeated on a 50 mL scale and the total volume was divided into two parallel reactions. These were composed of the following components: Tween® 20 (5 µL each) and diphosphatase (0.5 µL each) in HEPES buffer (22.7 mL). The GCoA solution (80 µL, 15.7 g/mol), derivative solution (36.7 mg in 4.36 mL NH_4HCO_3 (0.05 M), 250 µL) and finally $MgCl_2$ (2 M, 125 µL) were added and the reaction mixture was shaken (100 rpm) at 37 °C. Every 30 minutes a solution (250 µL) of derivative **9** was added up to a total volume of 2 mL per batch. After 2 h the GcoA-containing solution (80 µL each) was added. After the last batch was added, the reaction mixture was shaken (100 rpm) at 37 °C overnight. *n*-Pentane was added and the individual batches were combined. The phases were separated and the aqueous phase was extracted with *n*-pentane (3x) using ultrasound to accelerate separation. The combined organic phases were washed with brine, dried over $MgSO_4 \cdot H_2O$ and the solvent was removed *in vacuo*. To remove *n*-pentane residues the NMR sample was exposed to a stream of N_2 using a cold trap (−78 °C) located downstream. The crude product was purified by column chromatography (1. *n*-pentane : Et_2O= 5:1 then 2. *n*-pentane : MTBE= 2:1) to yield terpenoid **17** (1 mg) as a colorless oil.

$R_f = 0.15$ (*n*-pentane : MTBE= 5:1); ^{1}H NMR (600 MHz, C_6D_6): $\delta = 1.93$ – 1.88 (m, 1H, H_2), 1.72 – 1.63 (m, 3H, H_7, H_9, H_{12}), 1.61 – 1.54 (m, 2H, H_5, H_{10}), 1.53 – 1.45 (m, 3H, H_3, H_6, H_{10}), 1.41 – 1.36 (m, 1H, H_7), 1.33 – 1.29 (m, 1H, H_3), 1.23 – 1.19 (m, 3H, H_6, H_9, H_{11}), 0.99 – 0.91 (m, 8H, H_{11}, H_{12}, H_{14}, H_{15}), 0.78 (s, 3H, H_{13}) ppm; ^{13}C NMR (151 MHz, C_6D_6): $\delta = 73.1$ (C_8), 50.5 (C_{12}), 46.5 (C_5), 40.5 (C_7), 40.4 (C_2), 39.9 (C_9), 38.5 (C_{11}), 36.6 (C_3), 34.6 (C_4), 32.7 (C_1), 30.7 ($C_{14/15}$), 27.2 (C_{13}), 22.9 (C_6), 20.9 ($C_{14/15}$), 20.9 (C_{10}) ppm; HRMS [GC-MS, CI]: *m/z* calcd for $C_{15}H_{26}O$ $[M]^+$: 222.1984, found: 222.1976; $R_I = 1531$.

1.4 GC-MS data

Nine different STCs (BcBot2, GCoA, Tps32, Tri5, Cyc1, Hvs1, Cop4, PenA, Omp7) were probed on an analytical scale. GC-MS data of eight transformations revealed product formation of which only STCs GCoA and BcBot2 gave sufficient amounts of a major product. Thus, these two STCs were chosen for upscaling.

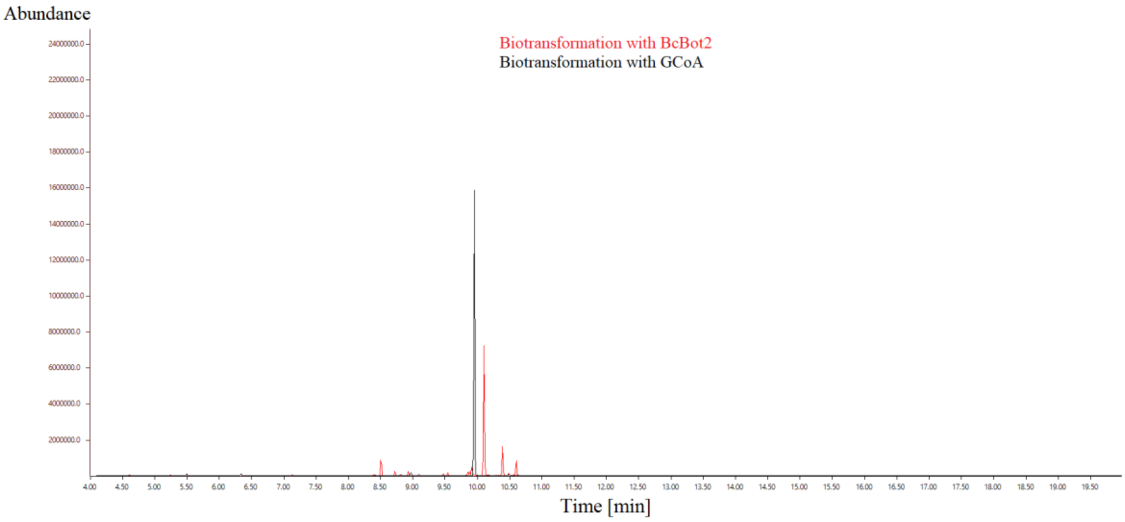

Figure S1. Overlay of the GC-data for the biotransformation of BcBot2 and GcoA with FPP derivative **9**.

For GCoA a single product with a *m/z*= 222 was found (see Figures S2 and S3).

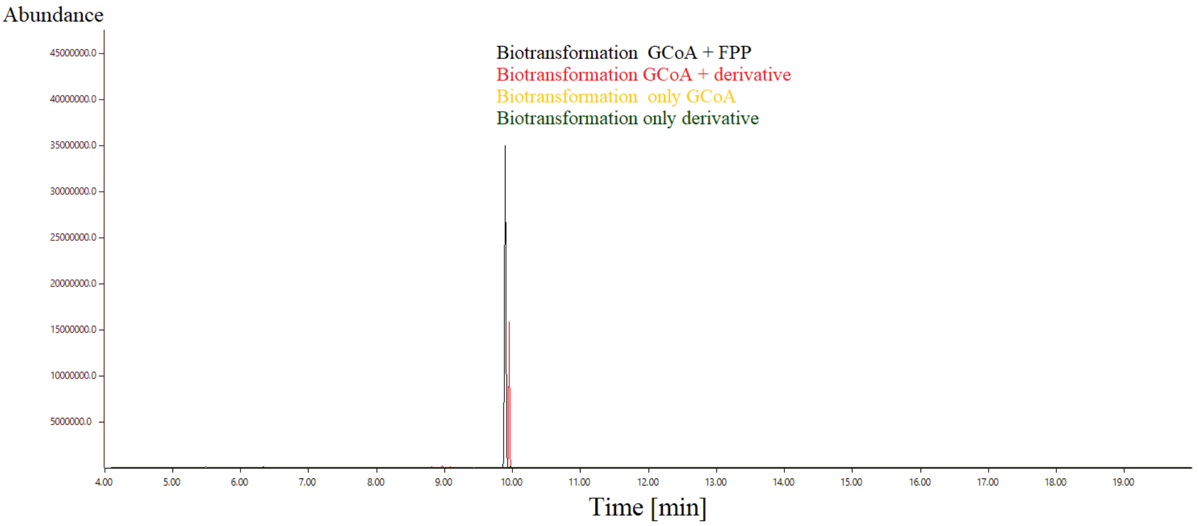

Figure S2. Overlay of the GC-data for the biotransformation of GCoA with FPP **1** and derivative **9**, including negative controls.

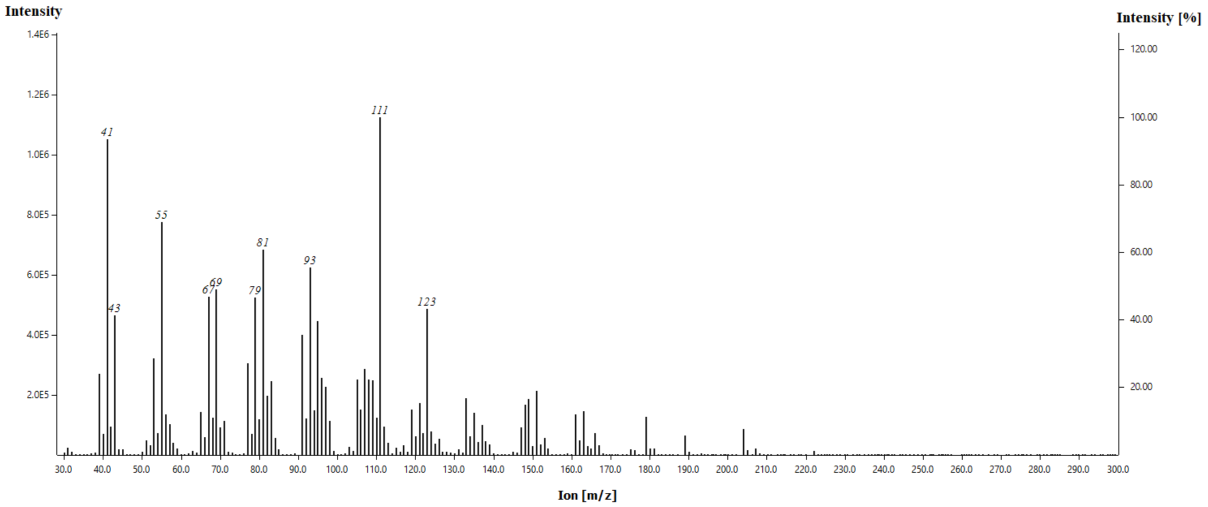

Figure S3. Mass spectrum for GC signal at 9.952 min obtained from the biotransformation of **9** with the GCoA.

In the case of BcBot2 a major product with a *m/z* value of 222 was formed (see Figures S4 and S5). The minor product was not further investigated in depth due to low signal intensity.

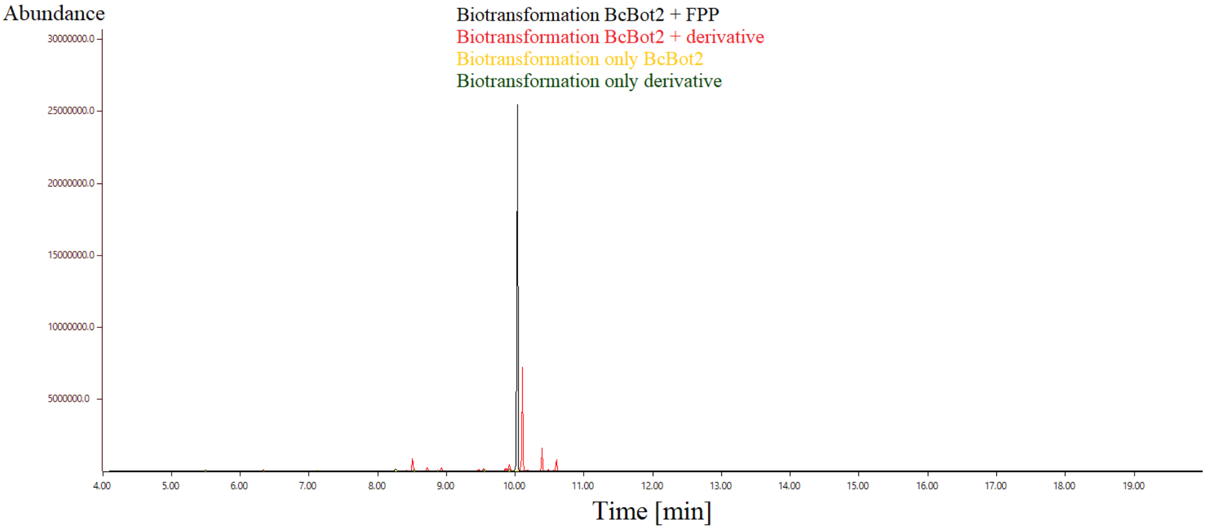

Figure S4. Overlay of the GC-data for the biotransformation of BcBOT2 with FPP **1** and derivative **9**, including negative controls.

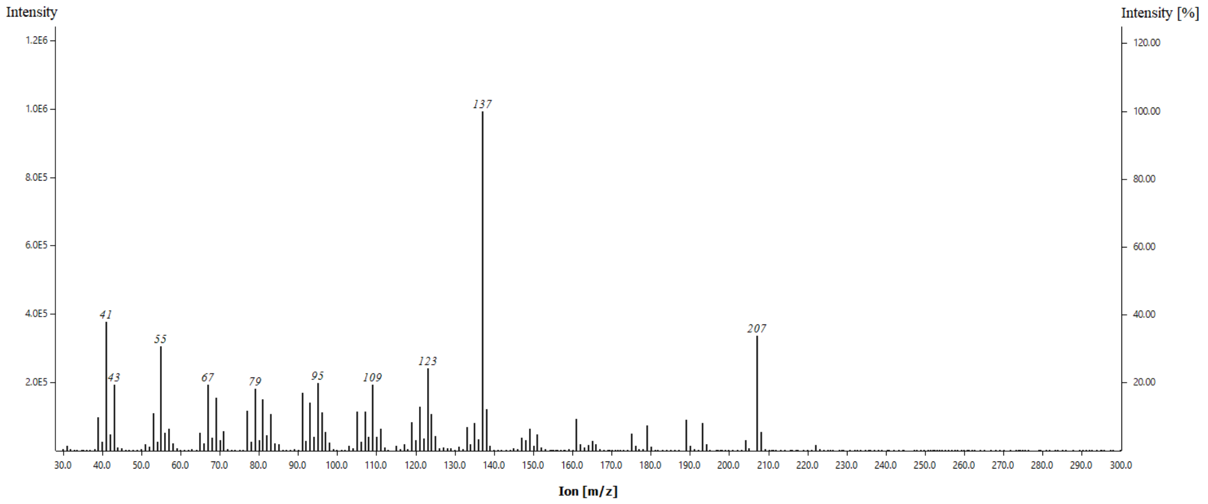

Figure S5. Mass spectrum for GC signal at 10.102 min obtained from the biotransformation of **9** with the BcBOT2.

1.5 Structure elucidation of product 16

Table S2. ^{1}H NMR signals and their corresponding ^{13}C NMR signals as analyzed by ^{1}H-^{13}C HSQC and ^{13}C{^{1}H} DEPT135 experiments for compound **16**. The quaternary carbon atoms are listed below.

δ (^{1}H)/ppm	δ (^{13}C)/ppm	DEPT135
1.99 + 1.76	48.3	CH_2
1.96 + 1.36	33.8	CH_2
1.78 + 1.68	42.3	CH_2
1.68	49.2	CH/CH_3
1.67 + 1.30	22.4	CH_2
1.51 + 1.33	42.0	CH_2
1.49 + 1.18	20.3	CH_2
1.41 + 1.25	58.3	CH_2
1.39	47.5	CH/CH_3
1.04	30.8	CH/CH_3
0.95	26.6	CH/CH_3
0.77	25.0	CH/CH_3
	73.7	C_{quart}
	39.5	C_{quart}
	35.8	C_{quart}

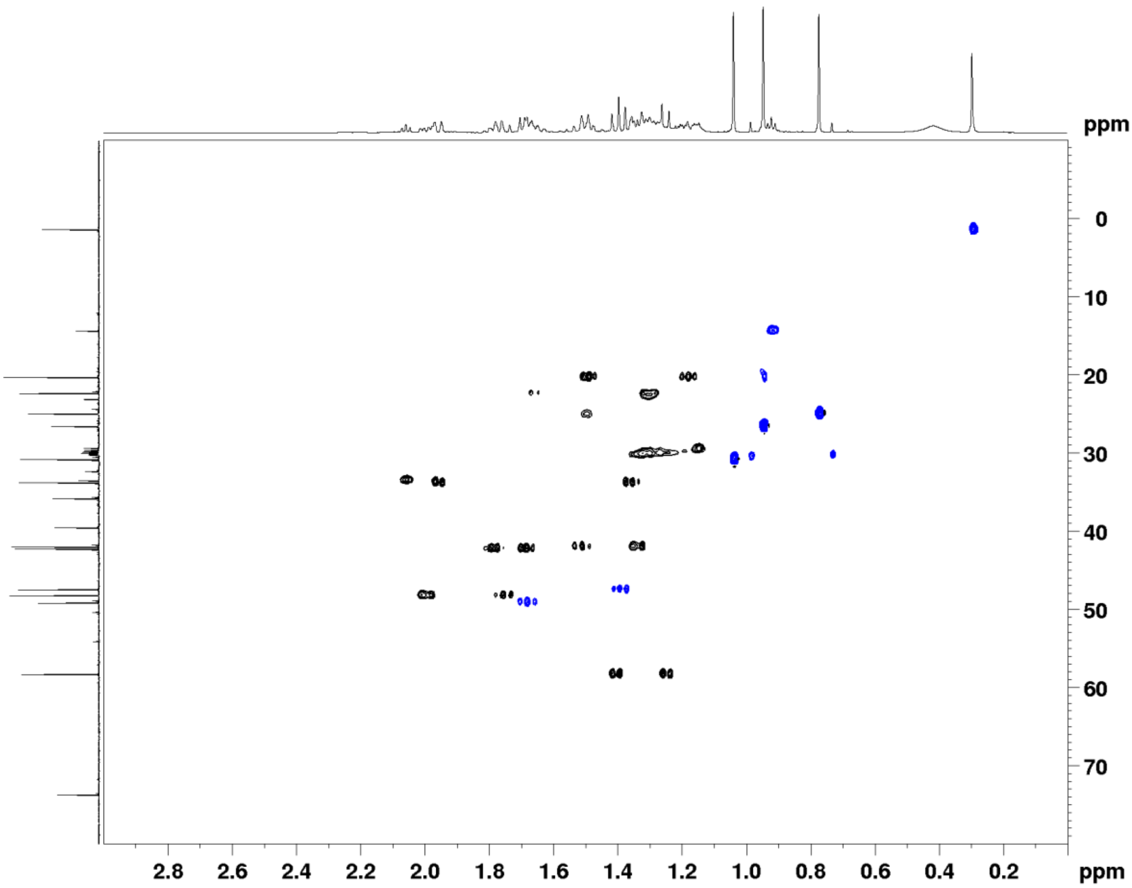

Figure S6. ^{1}H-^{13}C HSQC NMR spectrum of product **16** in C_6D_6 (pos. phase = blue (CH/CH_3), neg. phase = black (CH_2)).

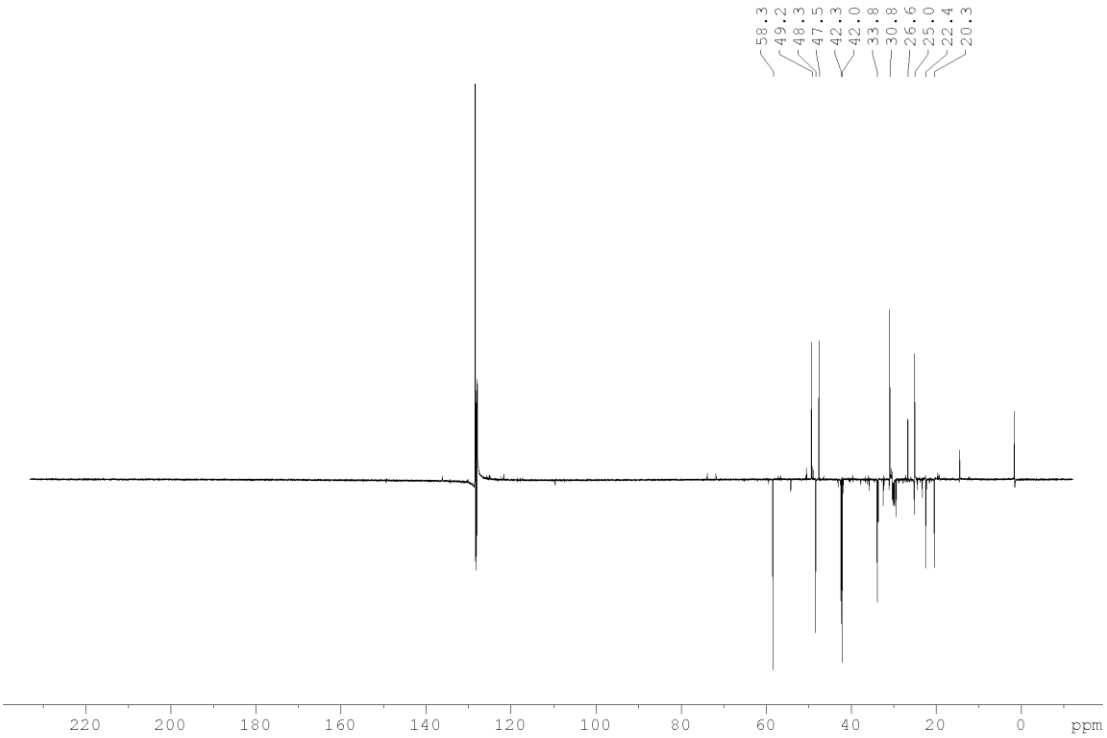

Figure S7. $^{13}C\{^{1}H\}$ DEPT135 NMR spectrum of product **16** in C_6D_6.

Table S3: ^{1}H NMR signals and their selected corresponding ^{1}H-^{1}H COSY correlation signals. Note: Signals are difficult to separate due to superposition and weak signals can be given in parentheses. If the exact ppm value cannot be deciphered due to superposition an x will be used to clarify this.

δ (^{1}H)/ppm	COSY correlations
1.99 + 1.76	1.30, 1.6x, each other
1.96 + 1.36	each other
1.78 + 1.68	1.18, 1.49, each other
1.68	1.39
1.67 + 1.30	1.99, 1.76 each other
1.51 + 1.33	0.95, 1.6x, each other
1.49 + 1.18	1.78, 1.68, each other
1.41 + 1.25	0.95, 0.77, each other
1.39	1.68
1.04	0.77
0.95	1.51, (1.41)
0.77	1.04, 1.41

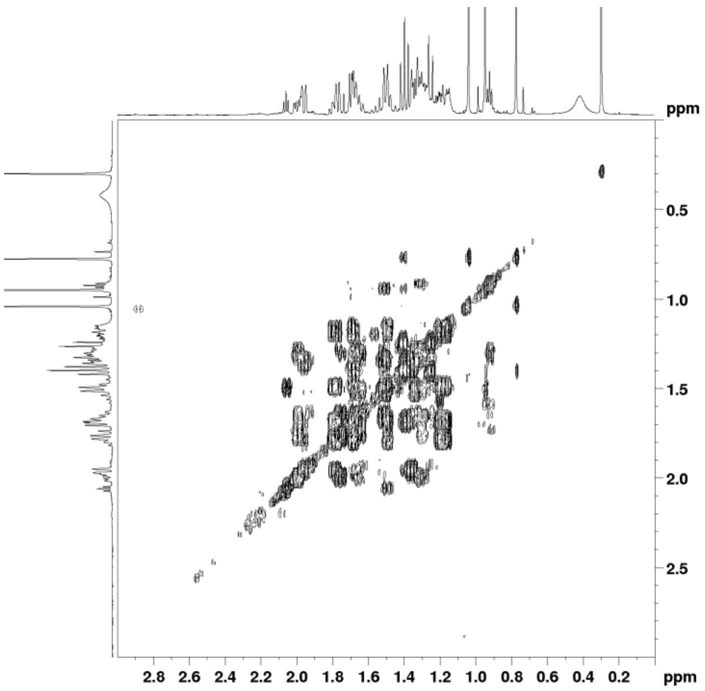

Figure S8. ^{1}H-^{1}H COSY NMR spectrum of compound **16** in C_6D_6.

Table S4: Selected ^{13}C NMR signals and neighbouring ^{1}H NMR signals obtained from the ^{1}H-^{13}C HMBC spectrum relevant for structure elucidation. Note: Weak signals are given in parentheses.

δ (^{13}C)/ppm	δ (^{1}H)/ppm
20.3	1.78 + 1.68
22.4	1.99 + 1.76, 1.33
25.0	1.68, 1.41 + 1.25, 1.04
26.6	1.51 + 1.33, 1.25, (1.39)
30.8	1.68, 1.41 + 1.25, 0.77
33.8	1.7x, 1.41/1.39 + 1.25
35.8	1.68, 1.41/1.39 + 1.25, 1.04, 0.77
39.5	1.96, 1.68, 1.51 + 1.33, 1.41 + 1.25, 0.95
42.0	1.99, 1.7x, 1.41 + 1.25, 0.95, (1.30)
42.3	1.96, 1.68, 1.7x, 1.49 + (1.18)
47.5	1.96, 1.68, 1.49 + 1.18, 1.41, 0.95
48.3	1.78, (1.6x)
49.2	1.96, 1.49 + 1.18, 1.39, 1.04, 0.77
58.3	1.51 + 1.33, 1.04, 0.95, 0.77
73.7	1.99, 1.96, 1.7x, 1.68, region of 1.5 to 1.3

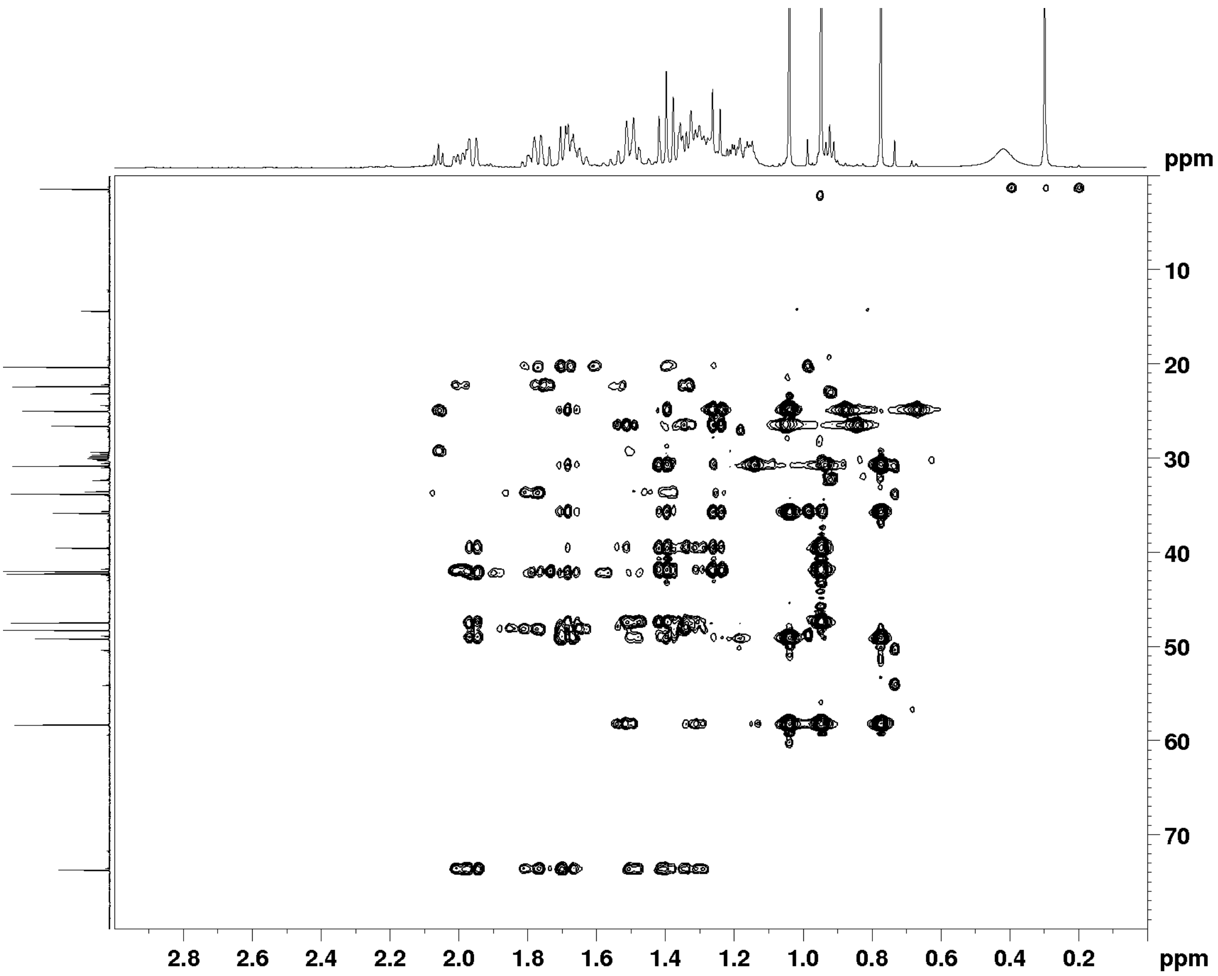

Figure S9. ^{1}H-^{13}C HMBC NMR spectrum of product **16** in C_6D_6.

To elucidate the configuration of product **16** ^{1}H-^{1}H-NOESY experiments were performed. The first issue to be addressed refers to the orientation of the vicinal CH groups. These can either be *syn* or *anti* orientated to each other. The NOESY spectra did not reveal crosspeaks between δ= 1.68 ppm and δ= 1.39 ppm, but instead between δ= 1.39 ppm and δ= 0.95 + 0.77 ppm and δ= 1.68 ppm and δ= 1.04 ppm. As a consequence the CH groups must be oriented *anti* to each other. From there also the absolute configuration of the product **16** can be deduced. The stereogenic center at δ= 39.5 ppm refers to the natural product presilphiperfolan-8-β-ol (**2**). The orientation of the hydroxyl group is set due to the lack of flexibility within the tricyclic core structure.

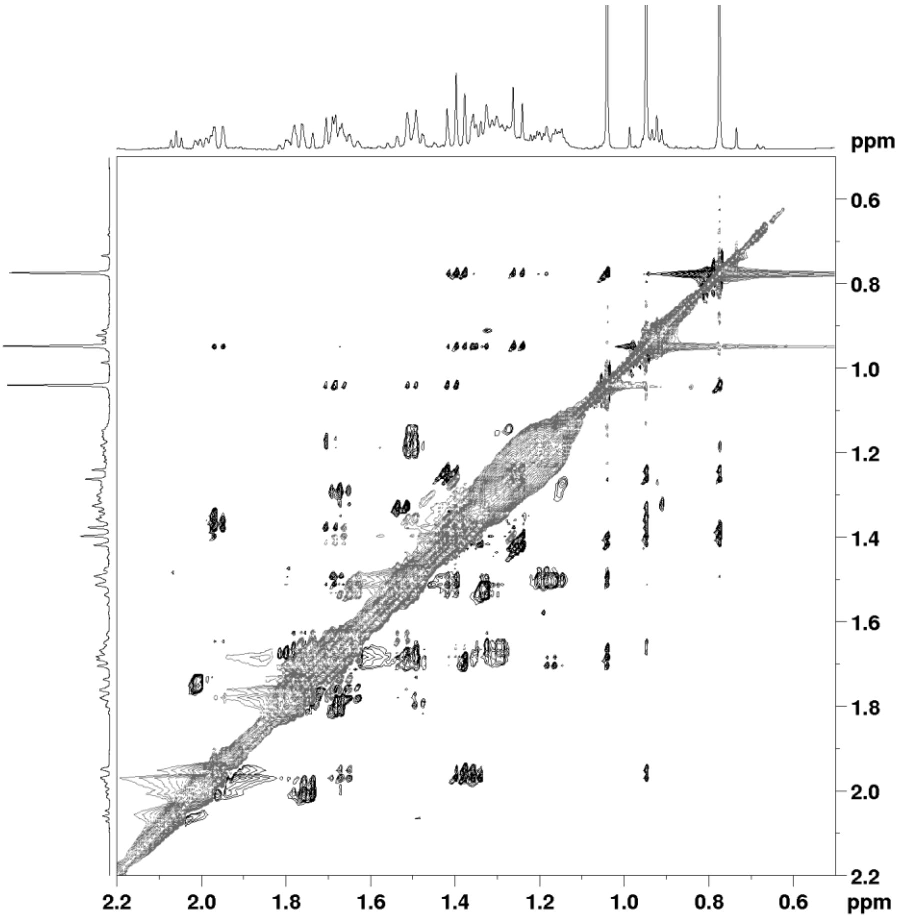

Figure S10. ^{1}H-^{1}H NOESY spectra of compound **16** in C_6D_6.

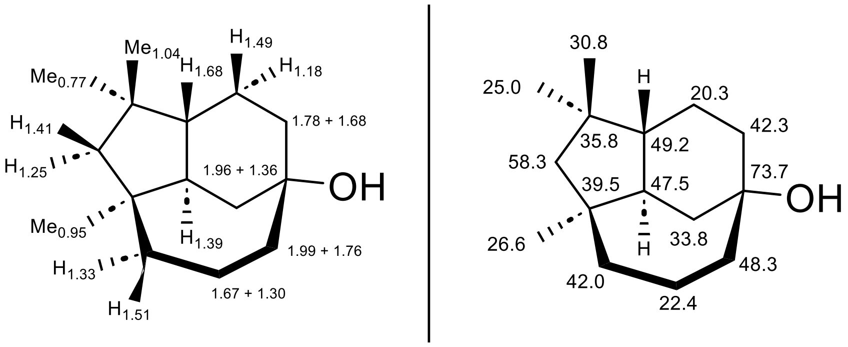

Figure S11. Fully assigned compound **16** including stereochemistry, δ (^{1}H)/ppm (le.) and δ (^{13}C)/ppm (ri.) values.

1.6 Structure elucidation of product 17

Table S5. ^{1}H NMR signals with their corresponding ^{13}C NMR signals as analyzed by ^{1}H-^{13}C HSQC and $^{13}C\{^{1}H\}$ DEPT135 experiments for isomer **17**. The quaternary carbon atoms are listed below.

δ (^{1}H)/ppm	δ (^{13}C)/ppm	DEPT135
1.91	40.4	CH/CH_3
1.69 + 1.38	40.5	CH_2
1.65 + 0.92	50.5	CH_2
1.64 + 1.21	39.9	CH_2
1.59 + 1.50	20.9	CH_2
1.55	46.5	CH/CH_3
1.50 + 1.20	22.9	CH_2
1.47 + 1.31	36.6	CH_2
1.21 + 0.95	38.5	CH_2
0.97	30.7	CH/CH_3
0.94	20.9	CH/CH_3
0.79	27.2	CH/CH_3
	73.1	C_{quart}
	34.6	C_{quart}
	32.7	C_{quart}

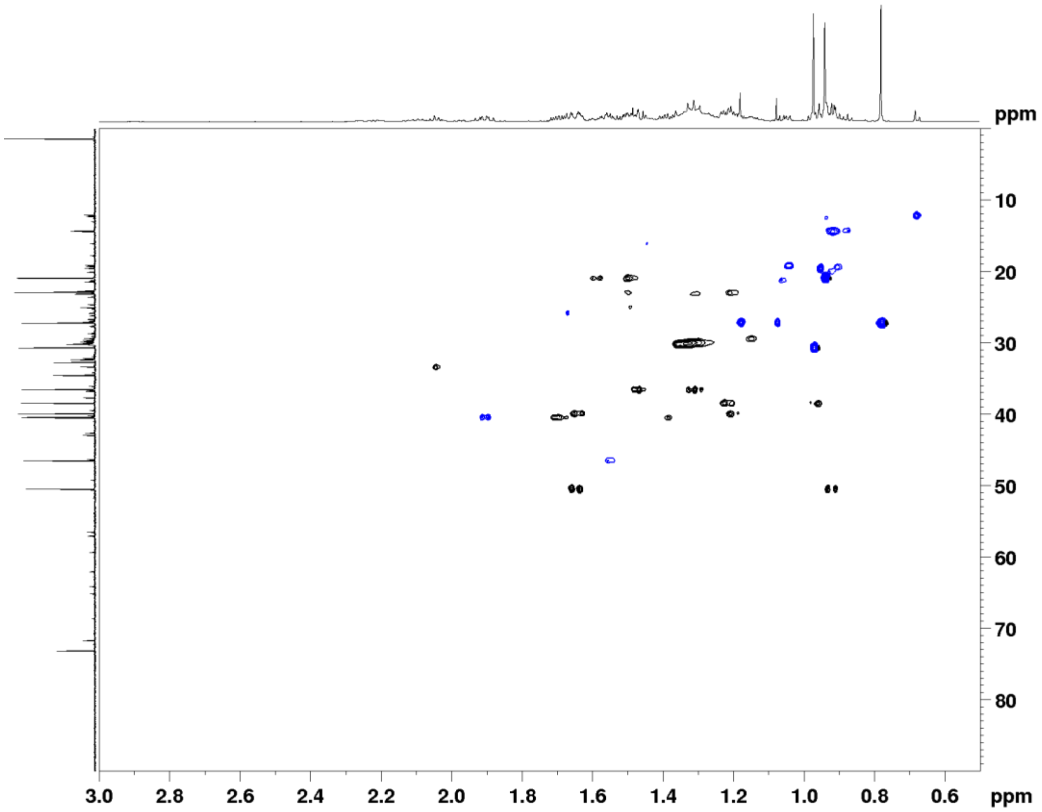

Figure S12. ^1H-^{13}C HSQC NMR spectrum of product **17** in C_6D_6 (pos. phase = blue (CH/CH_3), neg. phase = black (CH_2)).

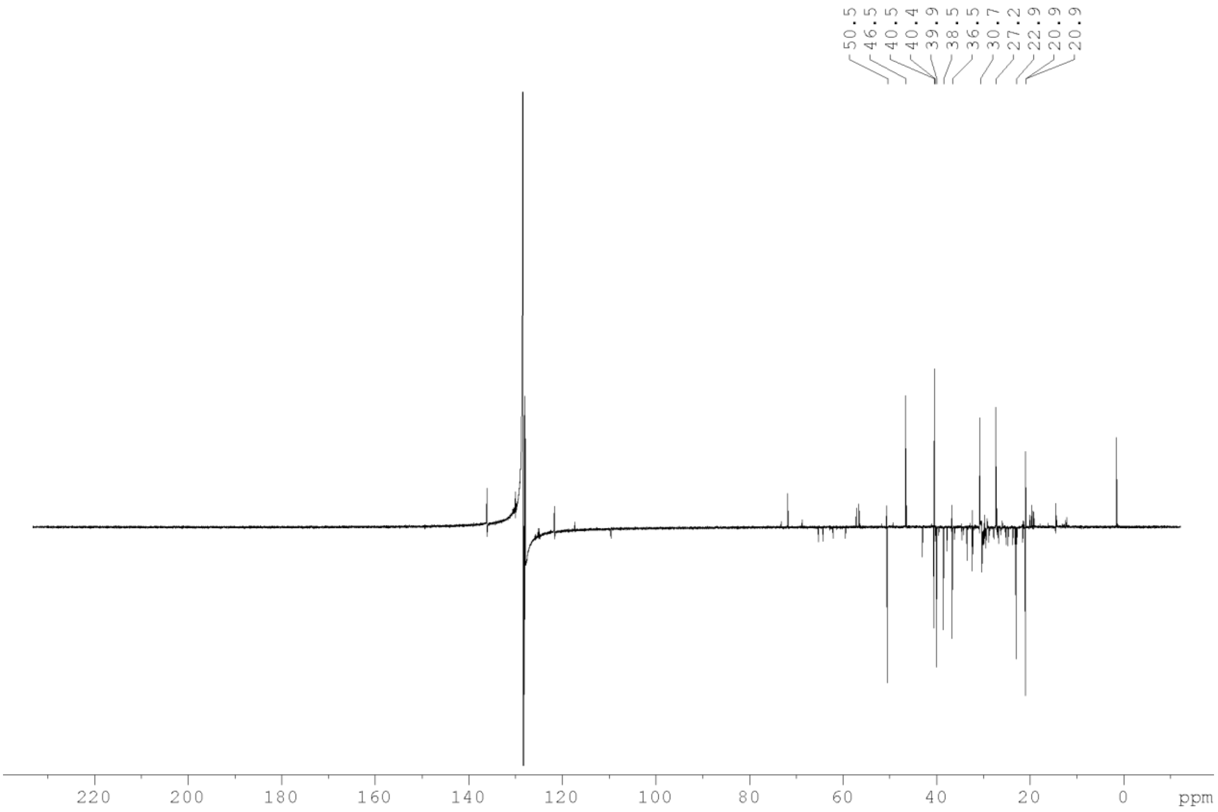

Figure S13. $^{13}C\{^1H\}$ DEPT135 NMR spectrum of product **17** in C_6D_6.

Table S6: ^{1}H NMR signals and their selected corresponding ^{1}H-^{1}H COSY correlation signals. Note: Signals are difficult to separate due to superposition and weak signals can be given in parentheses.

δ (^{1}H)/ppm	COSY correlations
1.91	1.31, 1.55, 1.47
1.69	1.20, 1.38, 1.50
1.65	0.92, 1.21
1.59	0.95, 1.21
1.55	0.97, 1.20, 1.91
1.50	1.69, 1.59, 1.38, 1.21, 0.95
1.47	1.91, 1.31
1.38	1.20, 1.50, 1.65, 1.69
1.31	0.92, 1.47, 1.91
1.21/1.20	0.95, 1.38, 1.65
0.97	
0.95	1.21, 1.50, 1.59
0.94	
0.79	(1.21, 1.65)

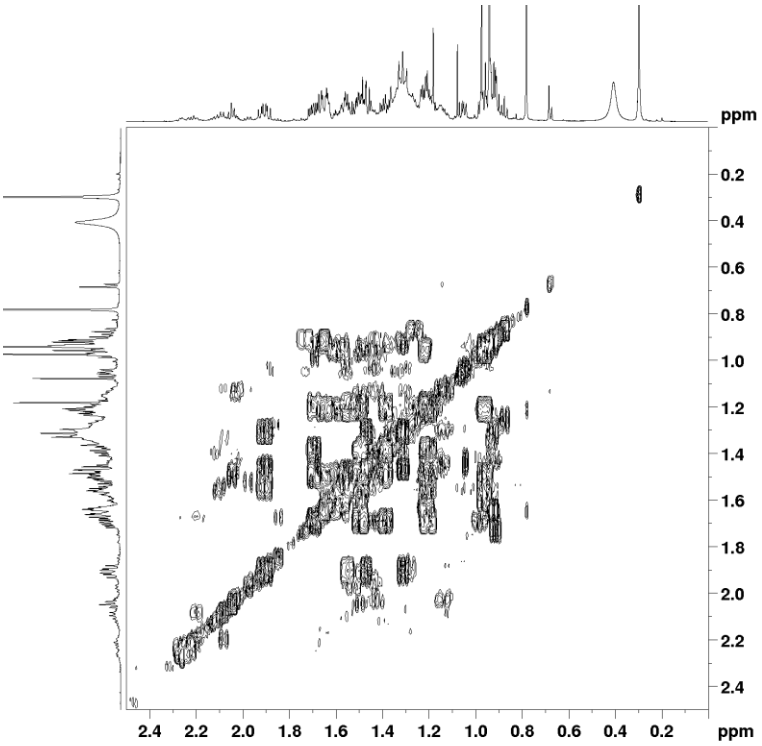

Figure S14. ^{1}H-^{1}H COSY NMR spectrum of isomer **17** in C_6D_6.

Table S7: Selected ^{1}H NMR signals and neighbouring ^{13}C NMR signals obtained from recording of the ^{1}H-^{13}C HMBC spectrum. Note: The 40.x ppm signals could not be clearly distinguished.

δ (^{1}H)/ppm	δ (^{13}C)/ppm
1.91	38.5, 22.9, 36.6, 46.5, 32.7, 27.2
1.65 + 0.92	73.1, 40.4, 39.9, 38.5, 32.7
1.50	40.x, 73.1
1.31 + 1.47	40.x, 34.6, 32.7, 30.7, 22.9, 20.9
region of 1.20/1.21	20.9, 40.x, 50.5, 73.1
0.97	20.9, 34.6, 36.6, 46.5
0.94	30.7, 34.6, 36.6, 46.5
0.92	40.x, 73.1
0.79	50.5, 40.x, 38.5, 32.7

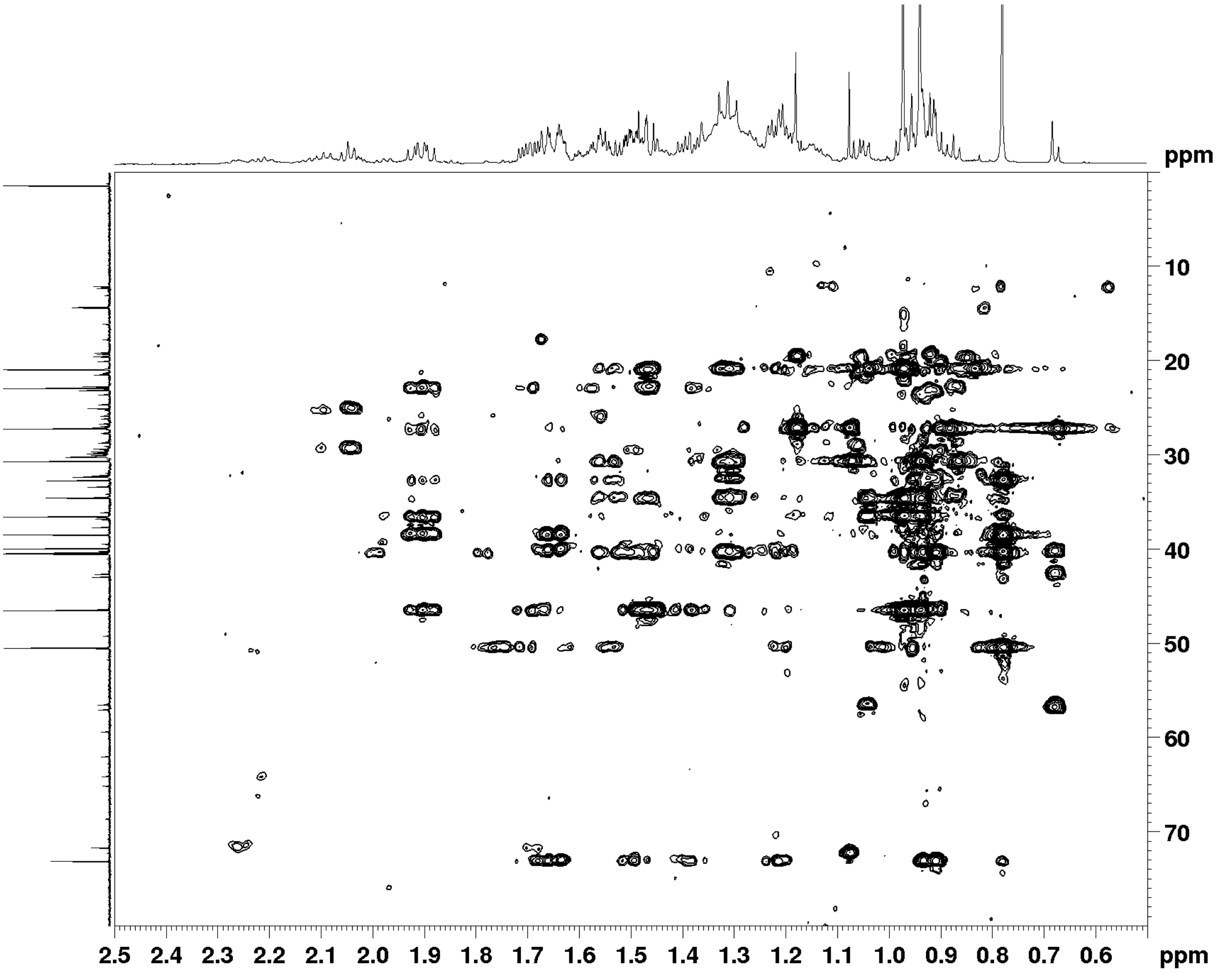

Figure S15. ^{1}H-^{13}C-HMBC NMR spectrum of product **17** in C_6D_6.

To elucidate the configuration of isomer **17** around the methylene bridge (**Figure S16**) ^{1}H-^{1}H-NOESY spectra were recorded.

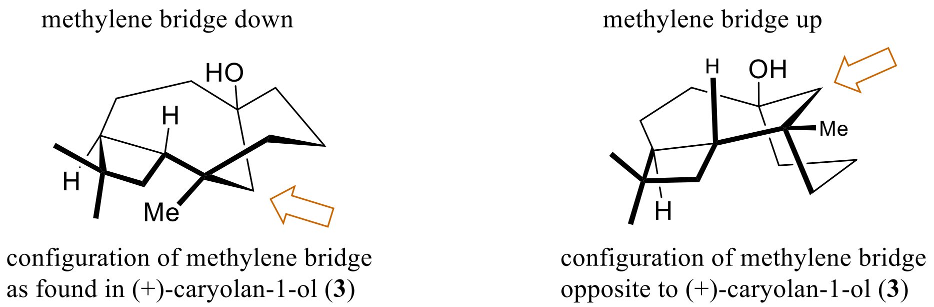

Figure S16. Diastereomers that may form according to the postulated mechanism.

The ^{1}H-^{1}H NOESY spectra clearly reveal that the methylene in **17** is located downwards just as found in the natural product (+)-caryolan-1-ol (**3**). This conclusion was drawn from the crosspeaks between 0.79 ppm (Me group) and 1.31, 0.79 and 1.55, 1.65 and 1.55 (weak) ppm lead to the conclusion of a downside bridge.

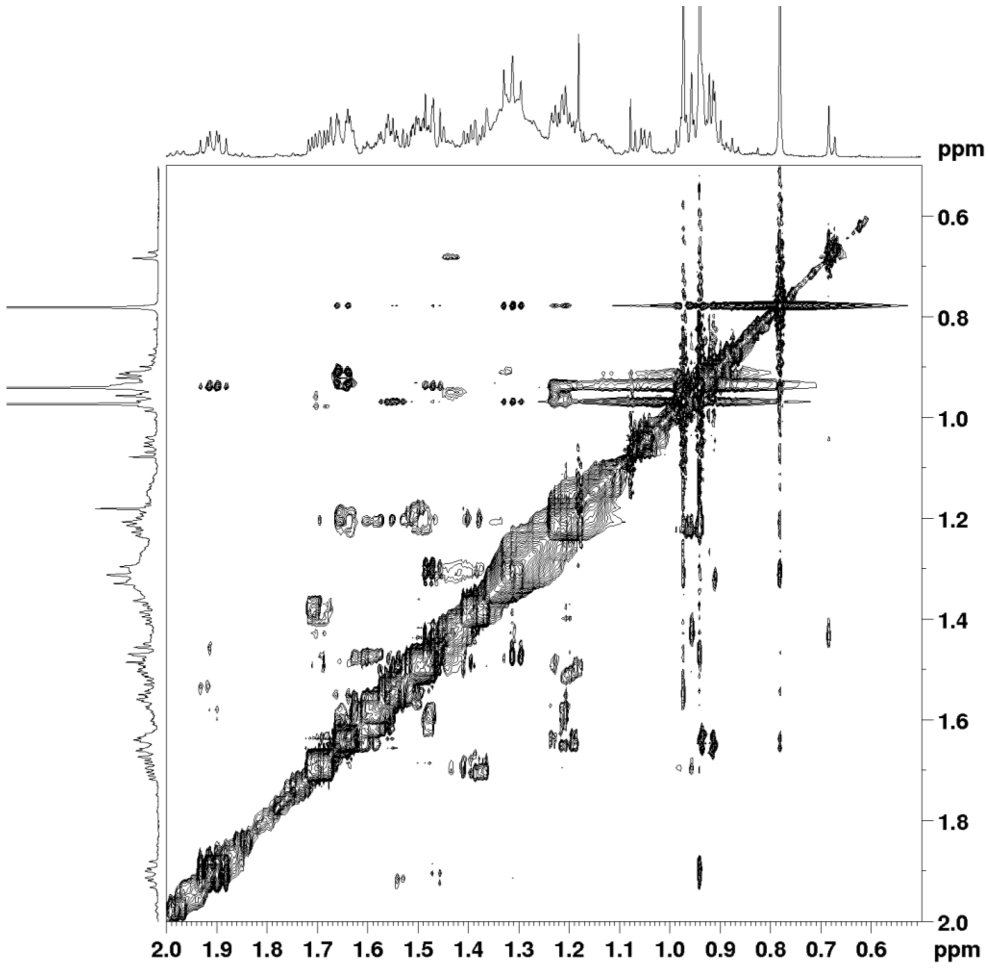

Figure S17. ^{1}H-^{1}H NOESY spectra of isomer **17** in C_6D_6.

Additionally, the 1D-NOE experiment focussed on δ= 1.54 ppm revealed a correlation with δ= 1.64 ppm confirming the conclusions drawn from the NOESY spectra.

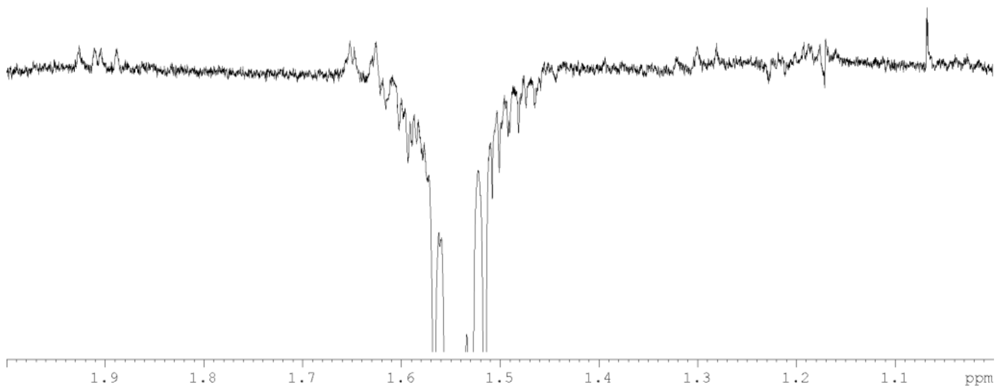

Figure S18. Excerpt of the1D-NOE spectra of isomer **17** with irradiation at δ= 1.54 ppm.

The absolute stereochemistry can be deduced from the natural product (+)-caryolan-1-ol (**3**) assuming that the formation of the cyclobutane ring proceeds in an identical manner for both pyrophosphates **1** and **9**. This applies to the stereogenic centers with protons at δ= 1.55 ppm and δ= 1.91 ppm.

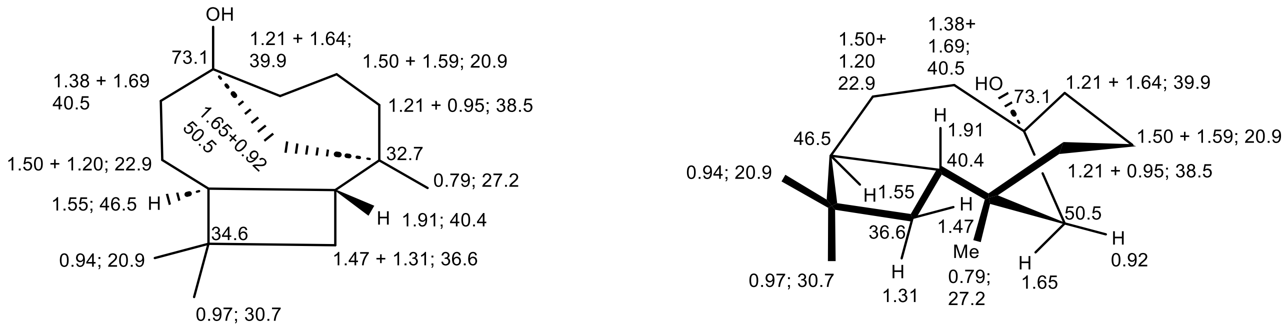

Figure S19. Fully assigned iso-caryolan-1-ol (**17**) including stereochemistry.

2. Copies of NMR spectra

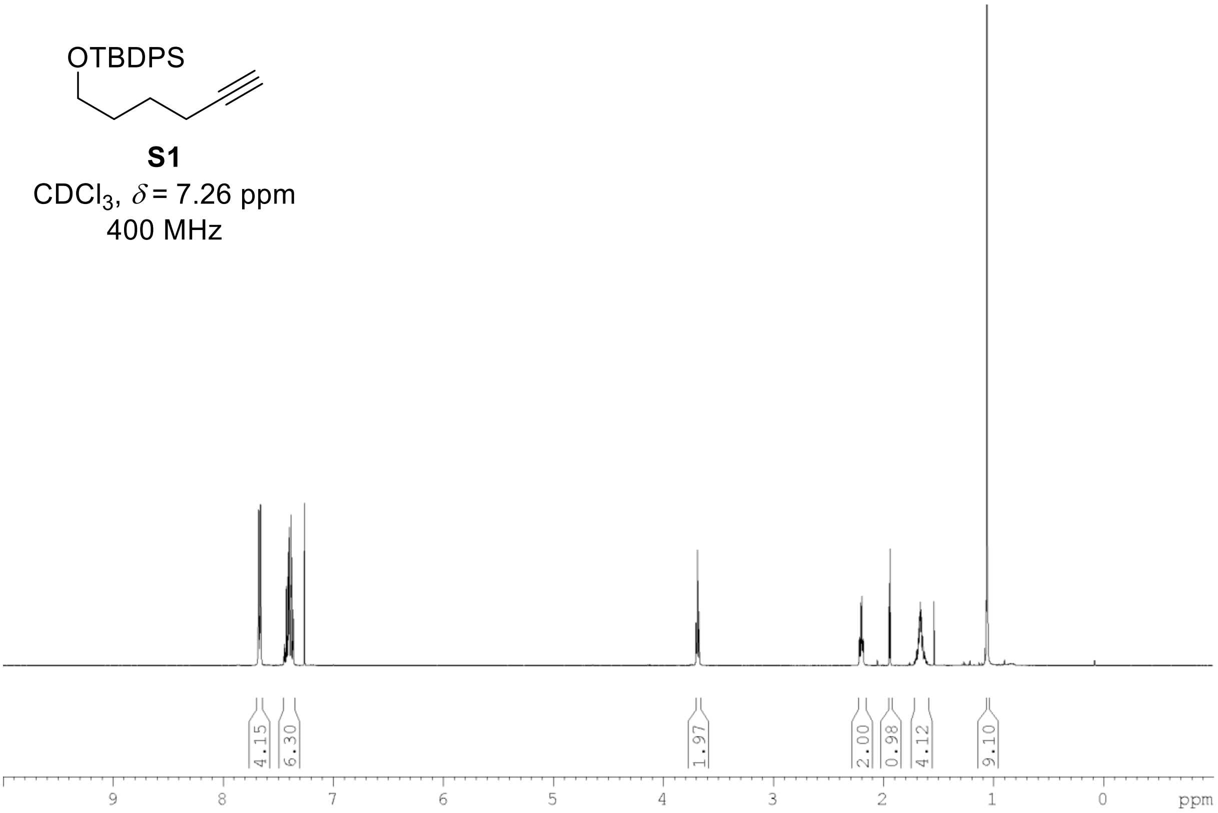

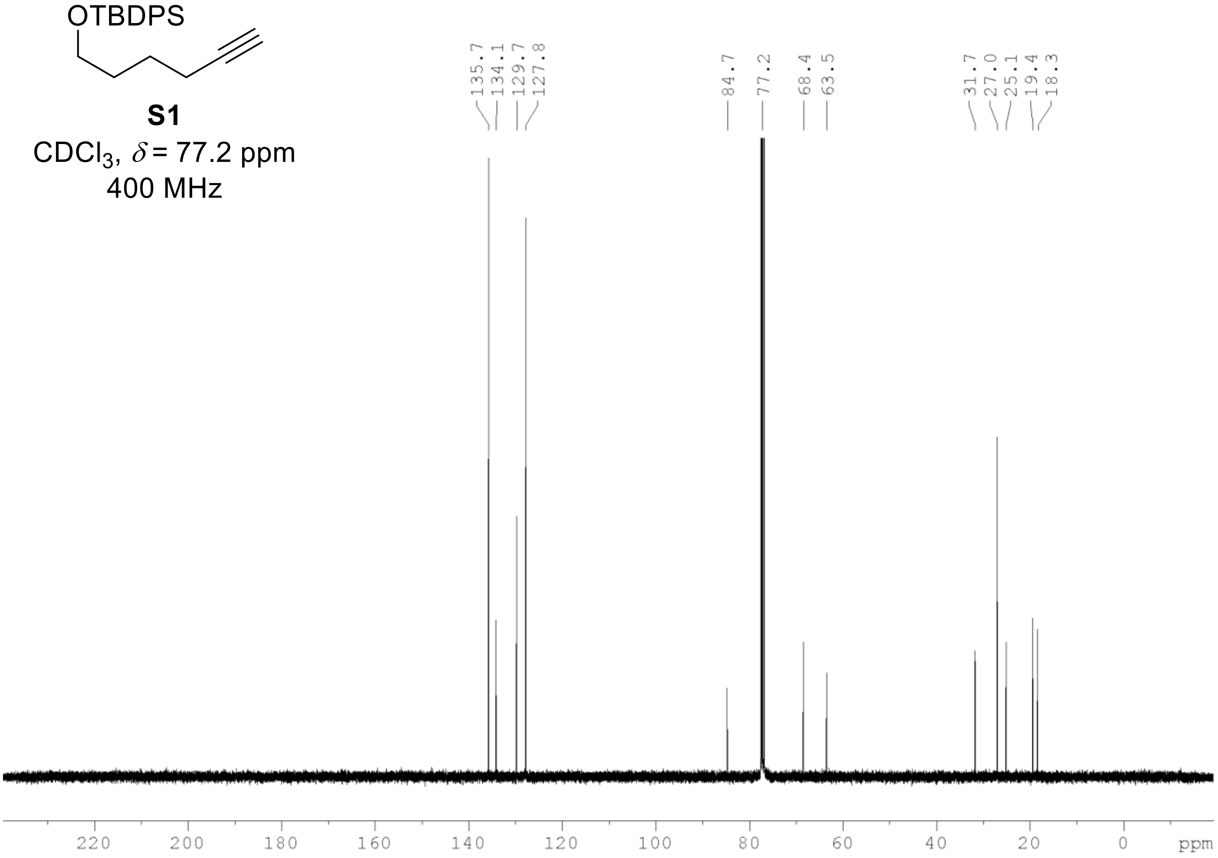

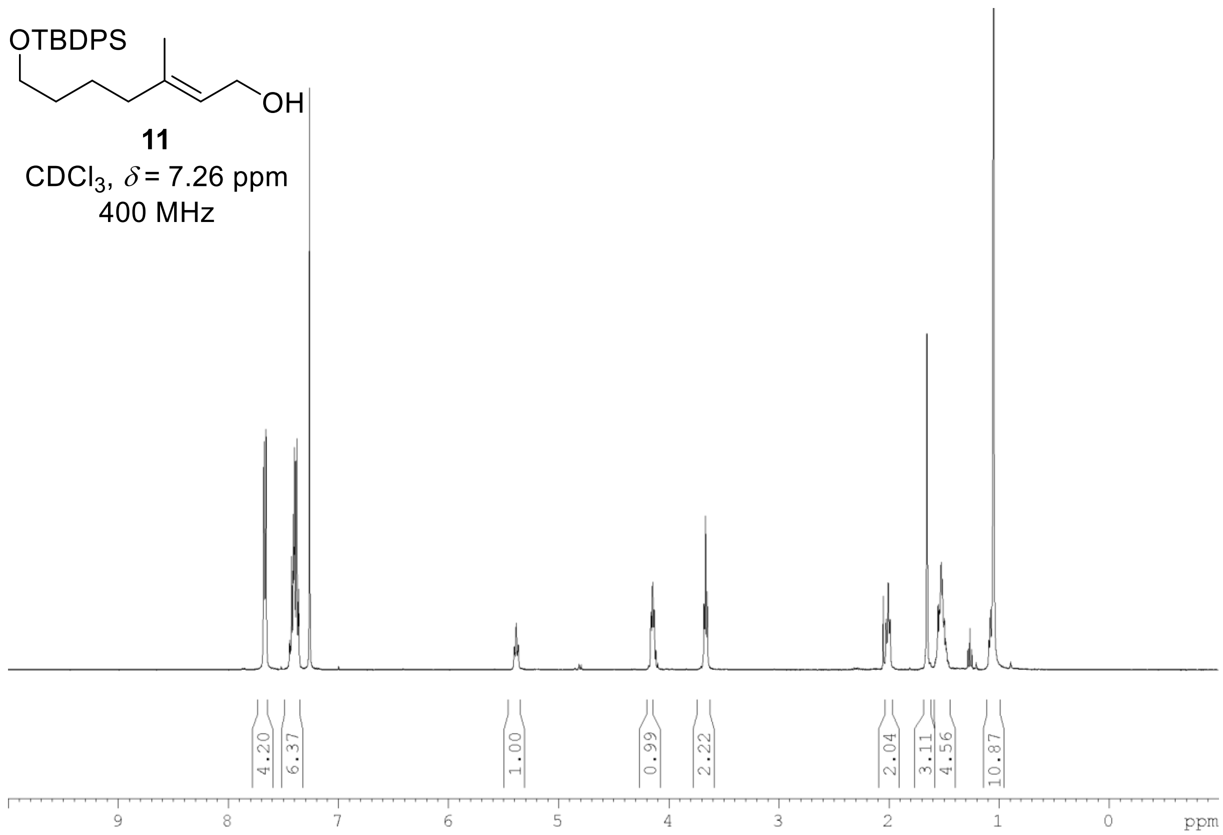
OTBDPS
OH
11
CDCl3, δ = 7.26 ppm
400 MHz
4.20
6.37
1.00
0.99
2.22
2.04
3.11
4.56
10.87
9
8
7
6
5
4
3
2
1
0
ppm

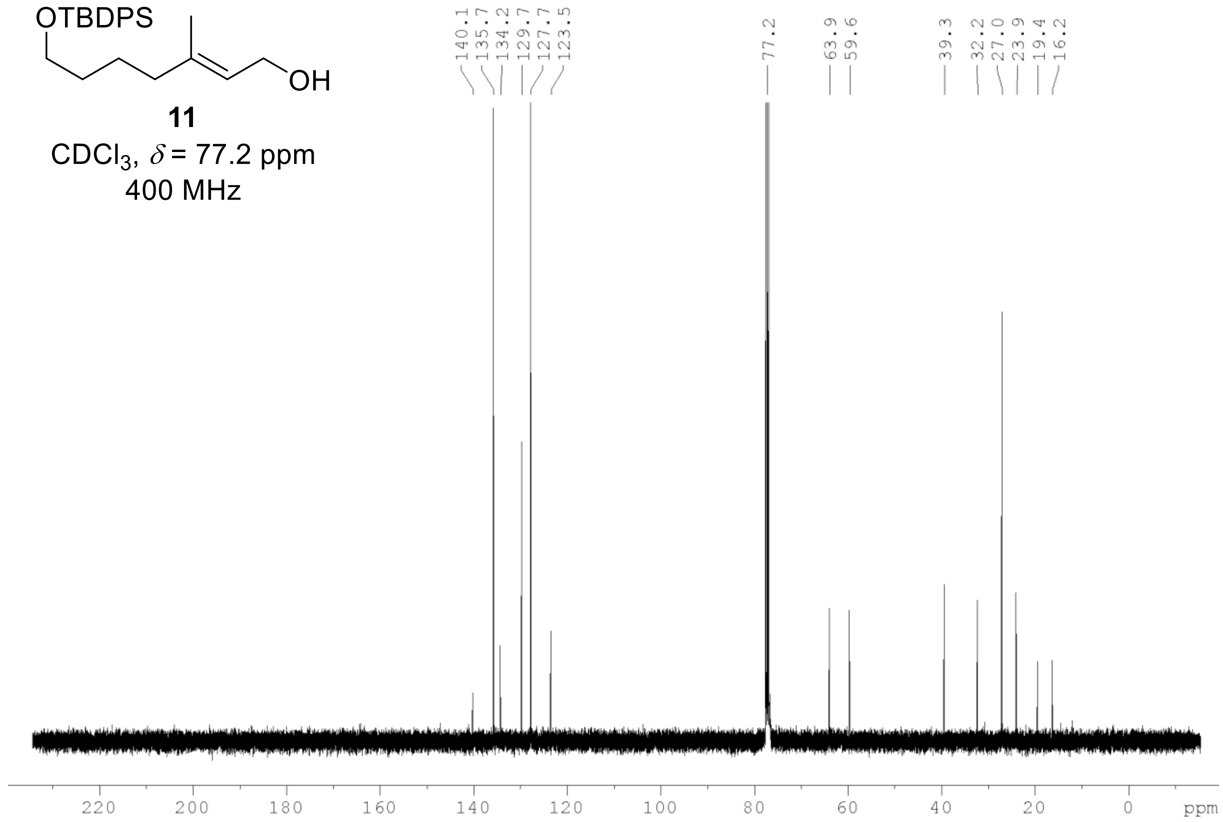
OTBDPS
OH
11
CDCl3, δ = 77.2 ppm
400 MHz
140.1
135.7
134.2
129.7
127.7
123.5
77.2
63.9
59.6
39.3
32.2
27.0
23.9
19.4
16.2
220
200
180
160
140
120
100
80
60
40
20
0
ppm

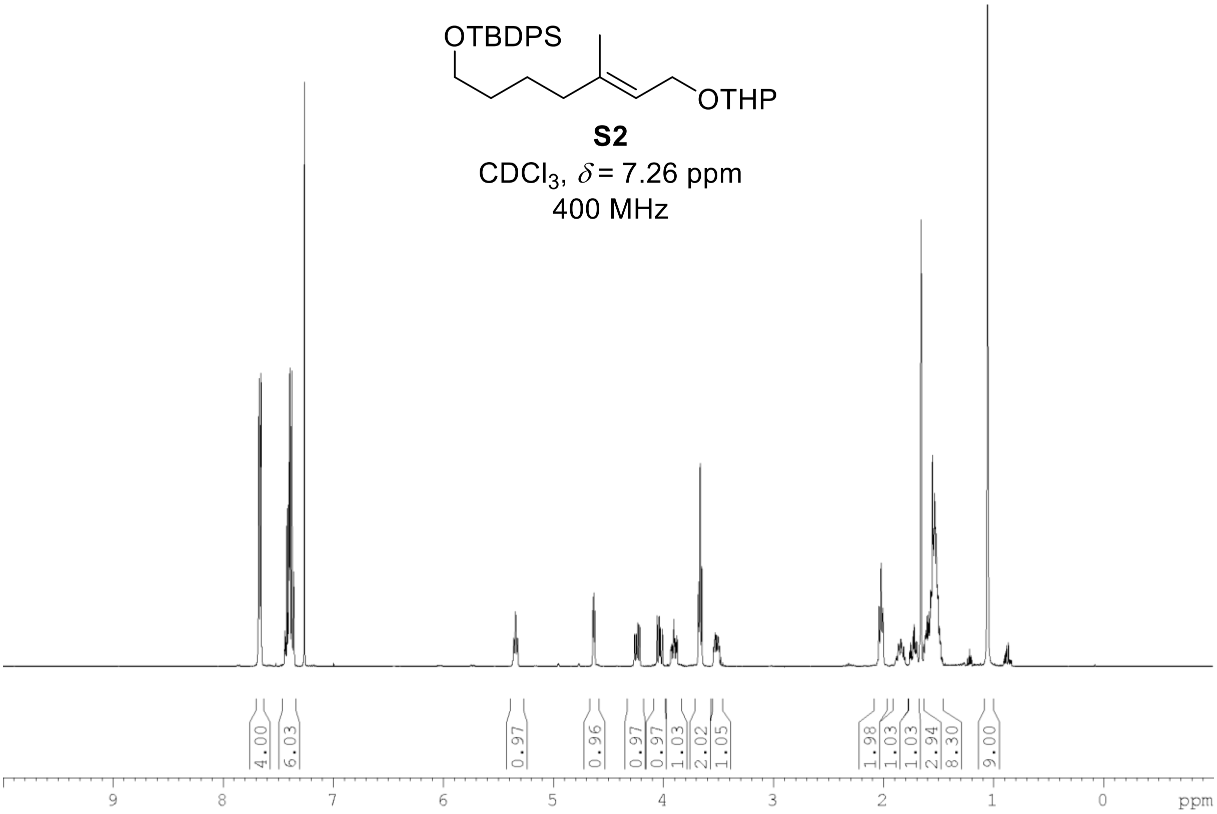
OTBDPS
OTHP
S2
CDCl3, δ = 7.26 ppm
400 MHz
4.00
6.03
0.97
0.96
0.97
0.97
1.03
2.02
1.05
1.98
1.03
1.03
2.94
8.30
9.00
9
8
7
6
5
4
3
2
1
0
ppm

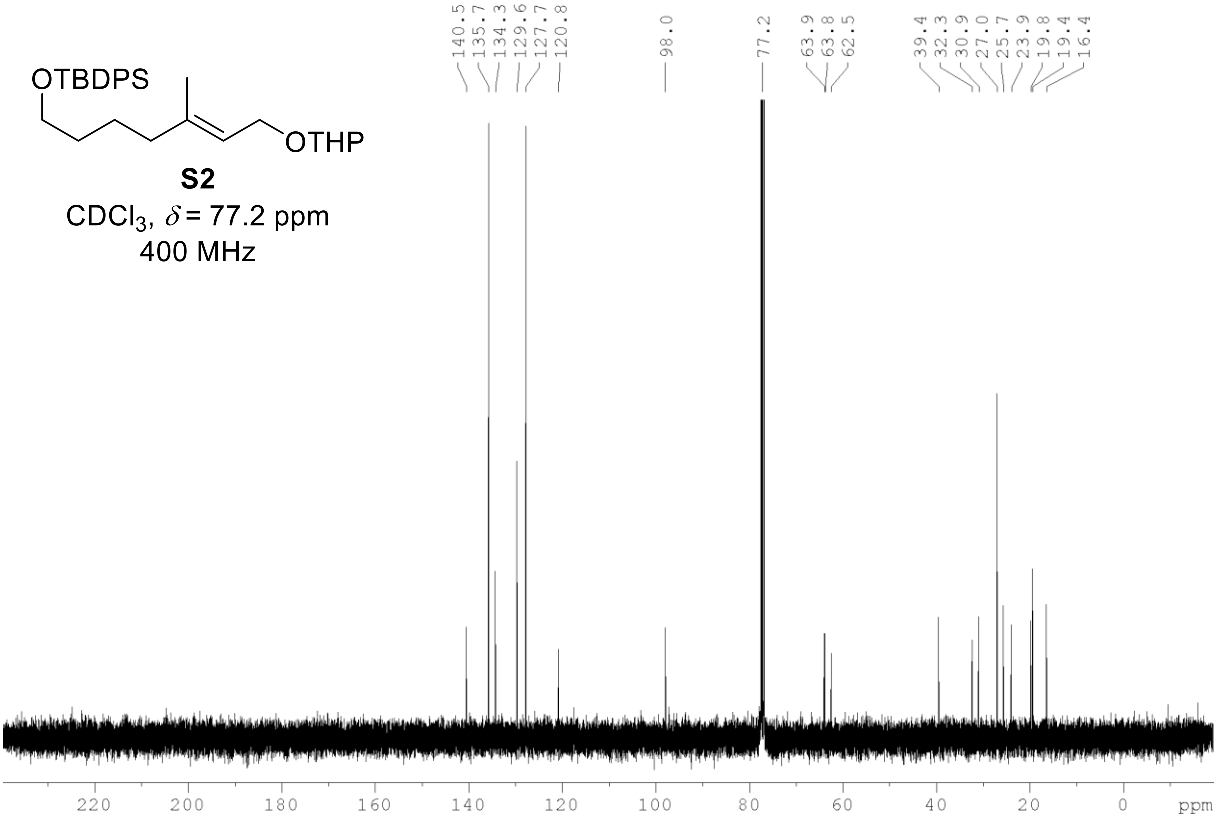
140.5
135.7
134.3
129.6
127.7
120.8
98.0
77.2
63.9
63.8
62.5
39.4
32.3
30.9
27.0
25.7
23.9
19.8
19.4
16.4
OTBDPS
OTHP
S2
CDCl3, δ = 77.2 ppm
400 MHz
220
200
180
160
140
120
100
80
60
40
20
0
ppm

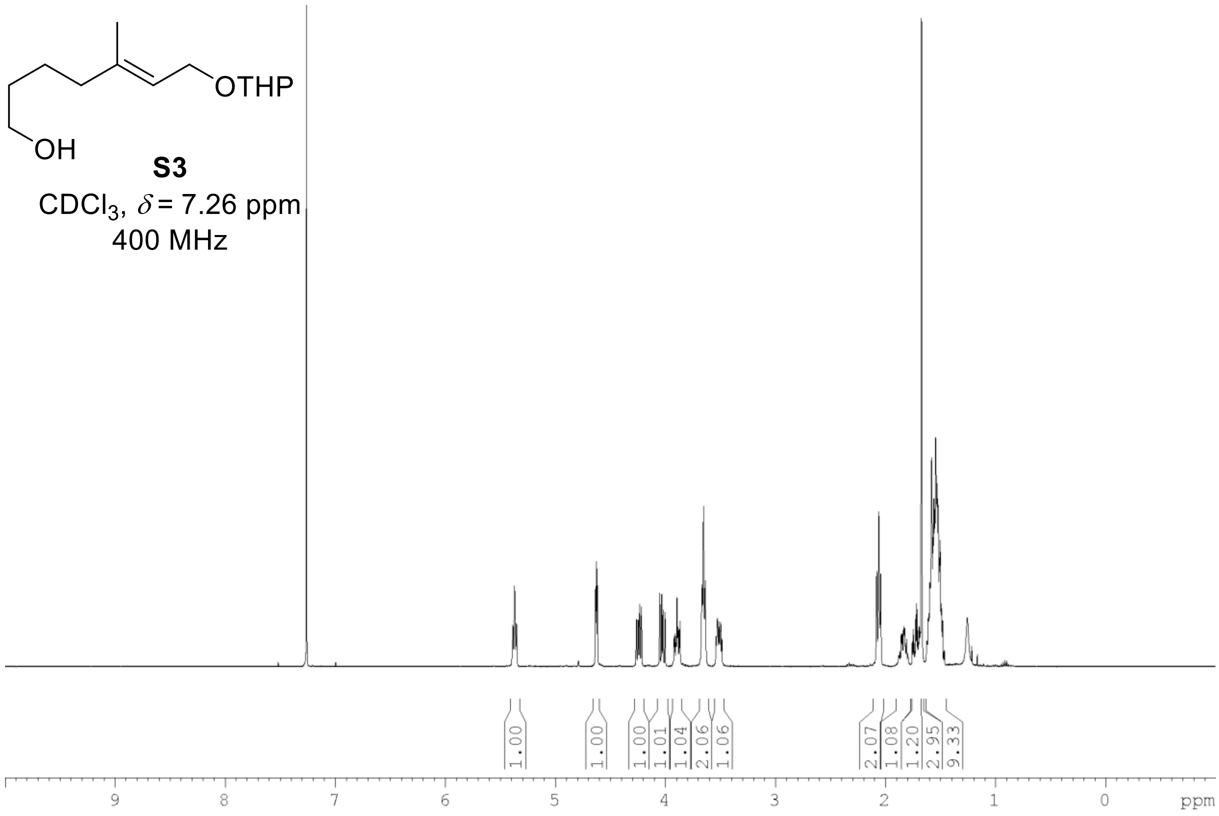
OTHP
OH
S3
CDCl3, δ = 7.26 ppm
400 MHz
1.00 1.00 1.00 1.01 1.04 2.06 1.06 2.07 1.08 1.20 2.95 9.33
9 8 7 6 5 4 3 2 1 0 ppm

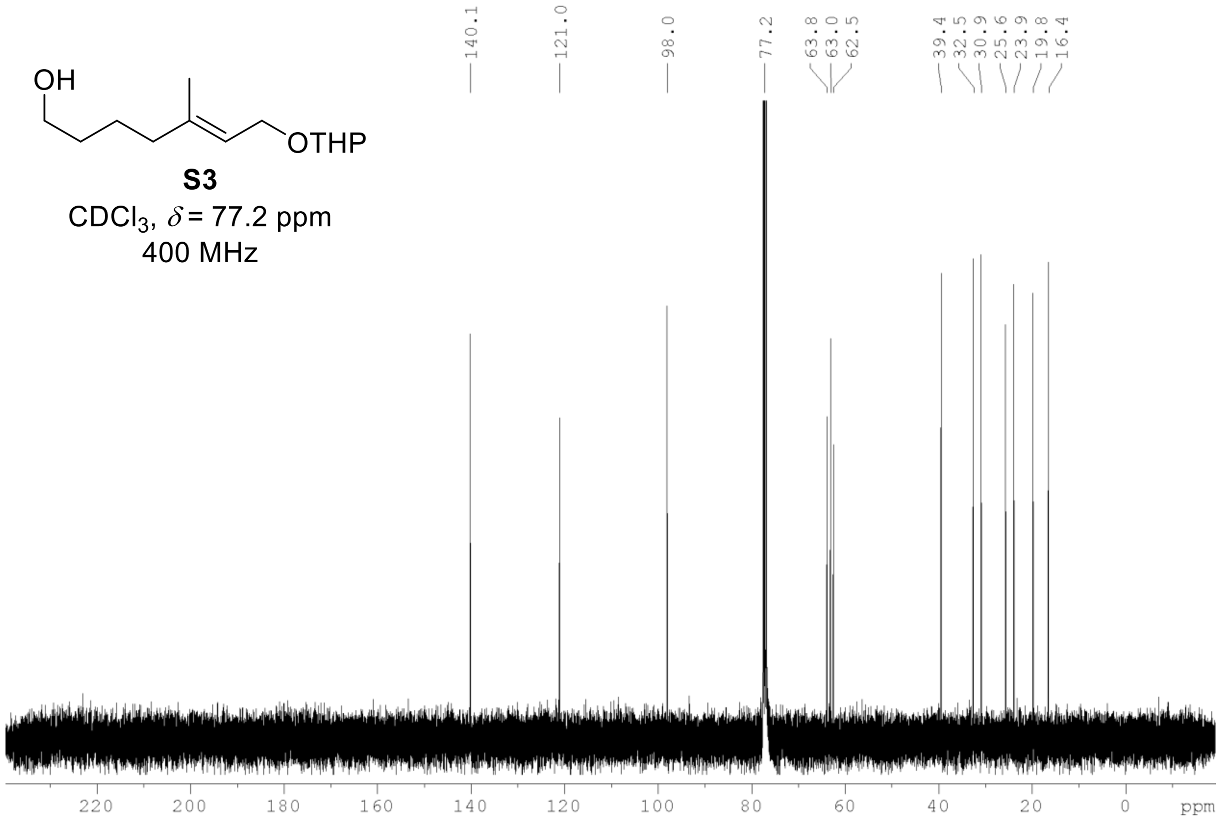
140.1 121.0 98.0 77.2 63.8 63.0 62.5 39.4 32.5 30.9 25.6 23.9 19.8 16.4
OH
OTHP
S3
CDCl3, δ = 77.2 ppm
400 MHz
220 200 180 160 140 120 100 80 60 40 20 0 ppm

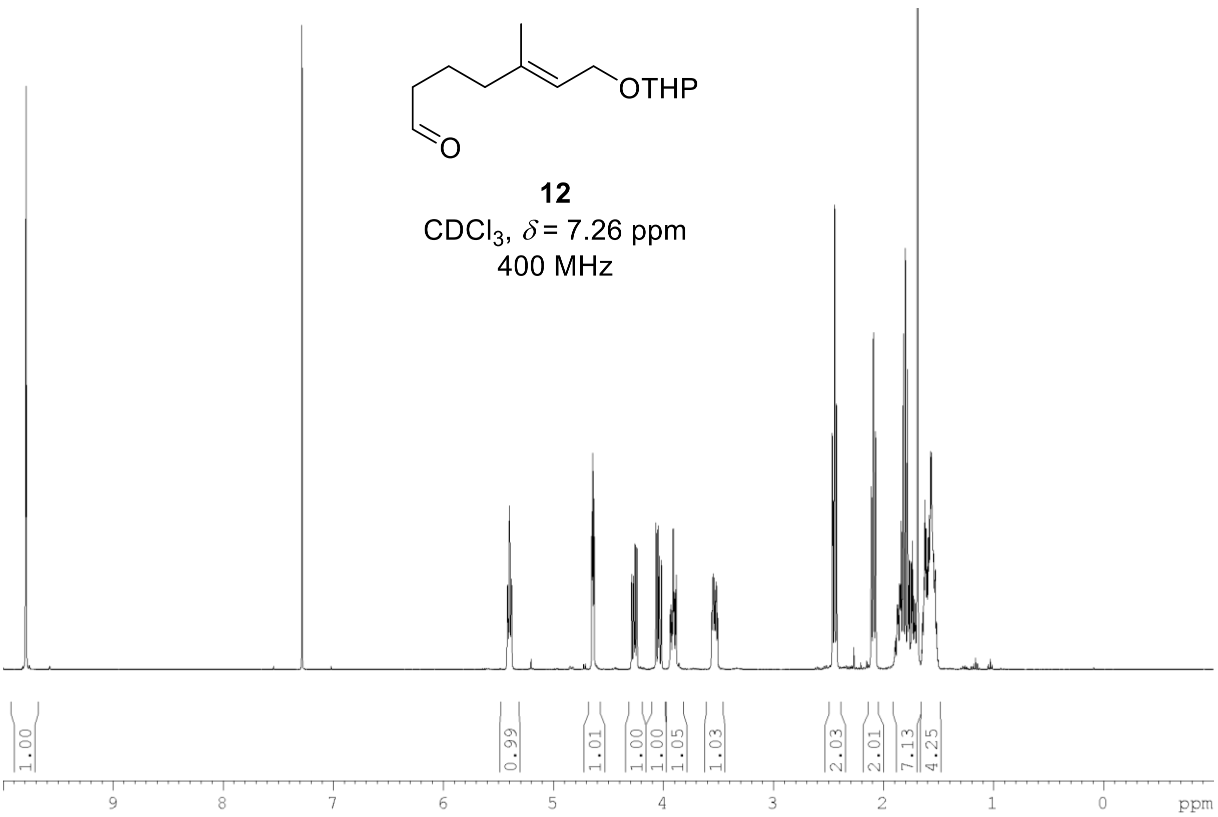
OTHP
O
12
CDCl$_3$, δ = 7.26 ppm
400 MHz
1.00
0.99
1.01
1.00
1.00
1.05
1.03
2.03
2.01
7.13
4.25
9
8
7
6
5
4
3
2
1
0
ppm

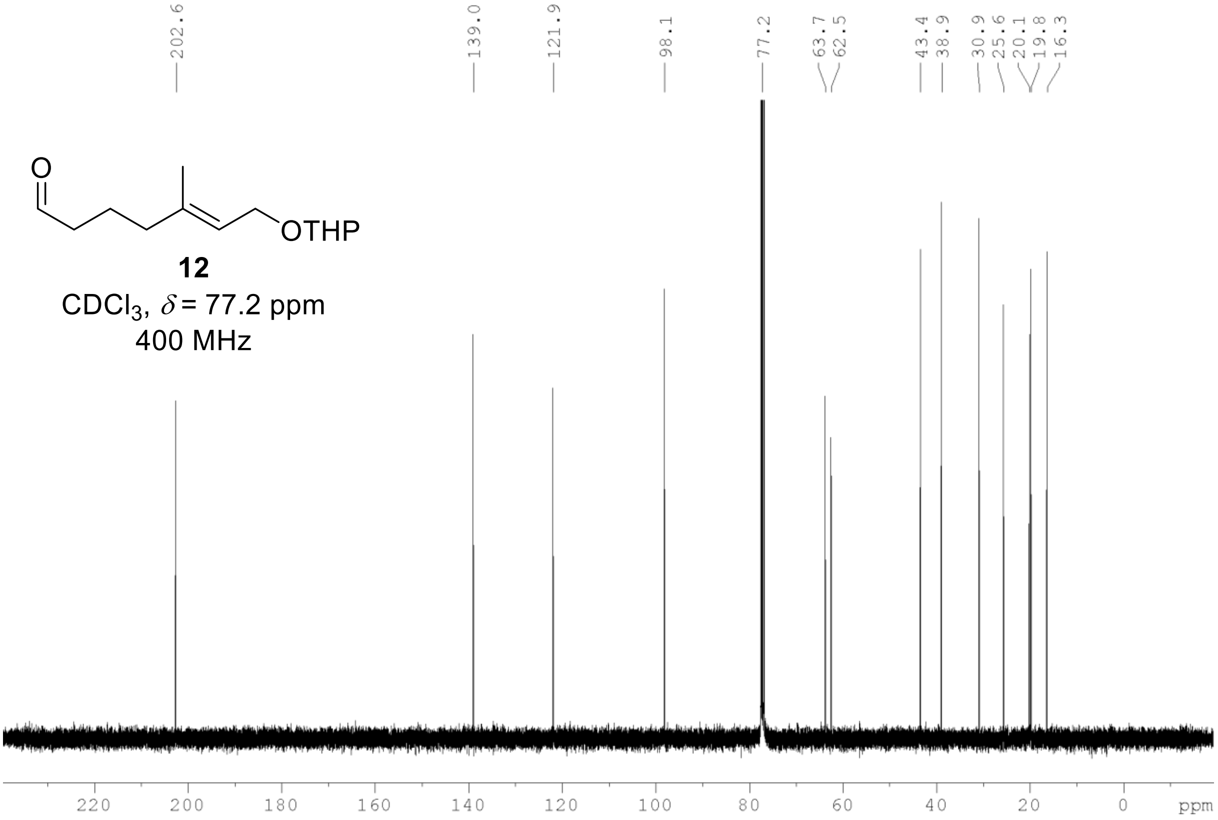
202.6
139.0
121.9
98.1
77.2
63.7
62.5
43.4
38.9
30.9
25.6
20.1
19.8
16.3
O
OTHP
12
CDCl$_3$, δ = 77.2 ppm
400 MHz
220
200
180
160
140
120
100
80
60
40
20
0
ppm

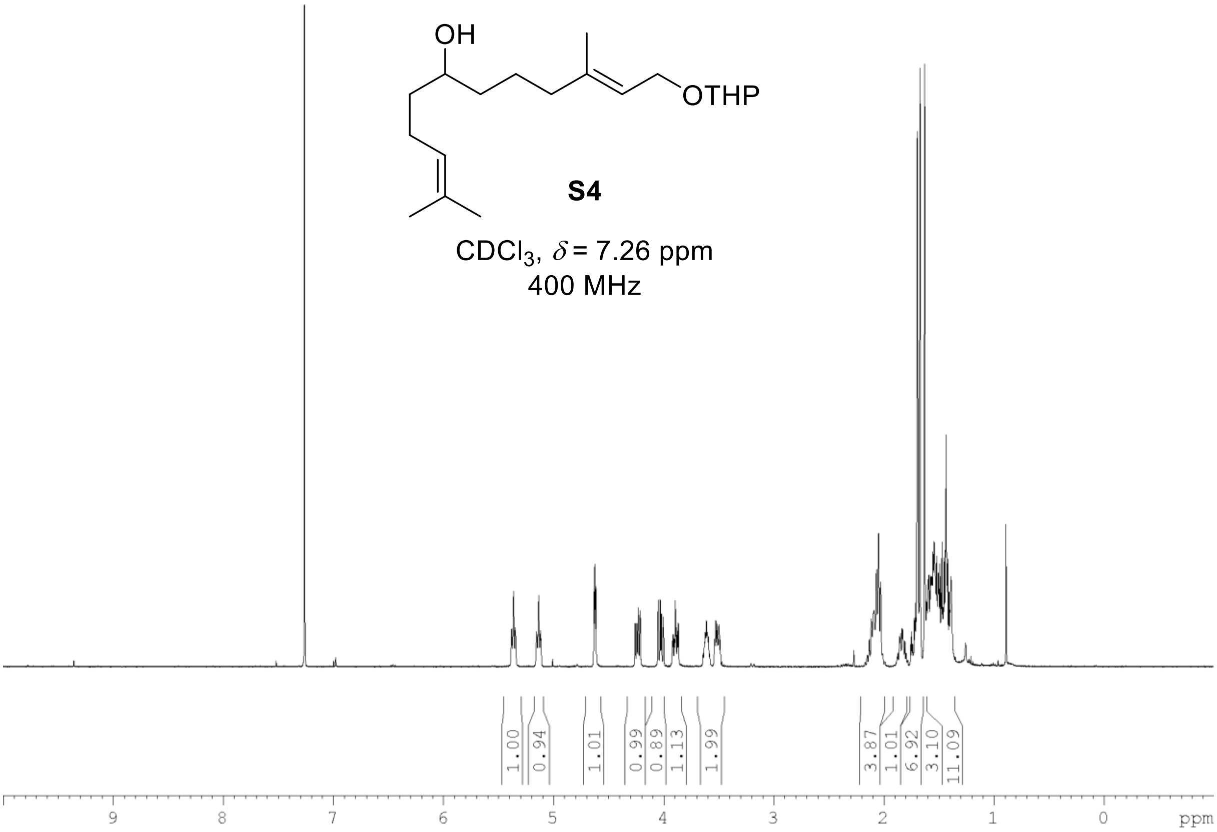
OH
OTHP
S4
CDCl3, δ = 7.26 ppm
400 MHz
1.00 0.94 1.01 0.99 0.89 1.13 1.99 3.87 1.01 6.92 3.10 11.09
9 8 7 6 5 4 3 2 1 0 ppm

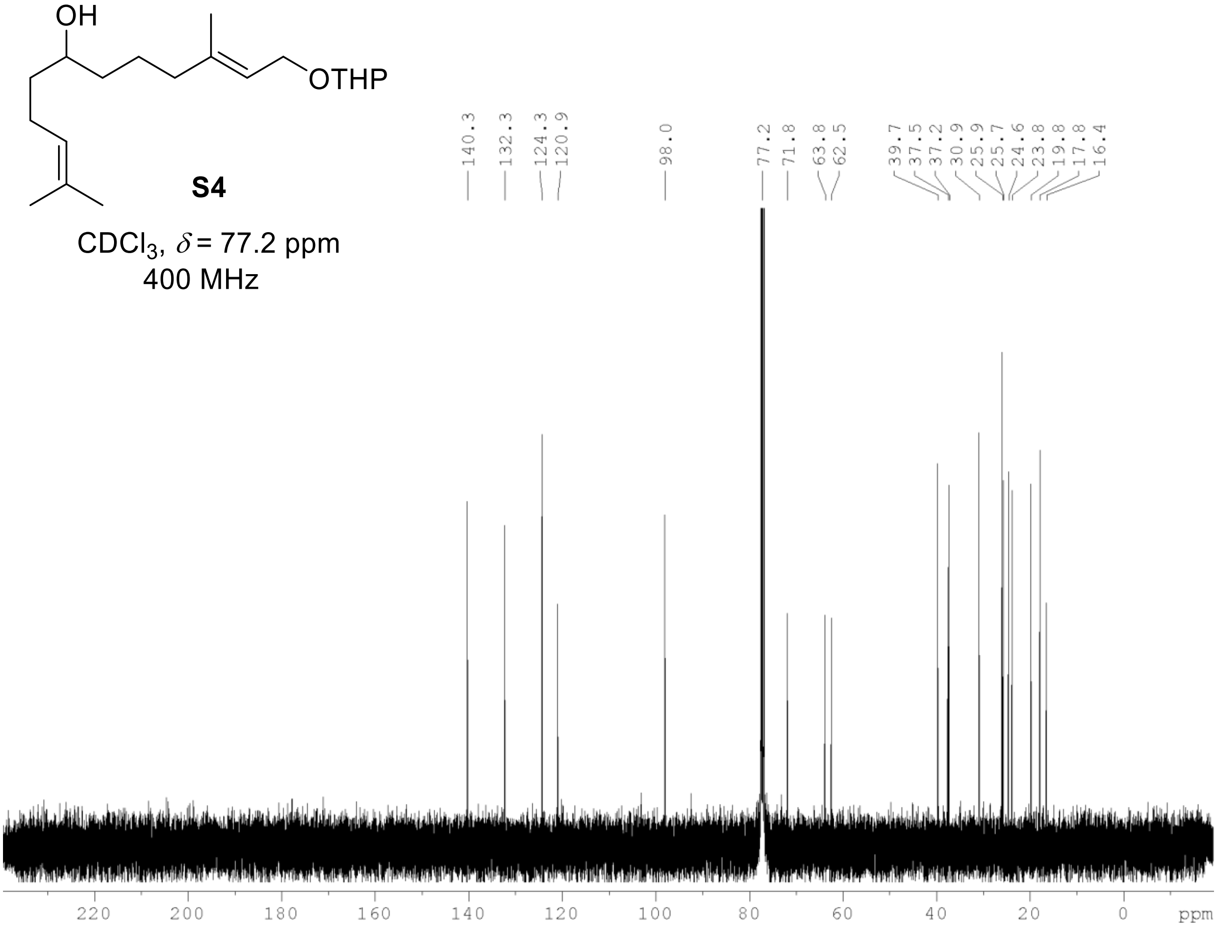
OH
OTHP
S4
CDCl3, δ = 77.2 ppm
400 MHz
140.3 132.3 124.3 120.9 98.0 77.2 71.8 63.8 62.5 39.7 37.5 37.2 30.9 25.9 25.7 24.6 23.8 19.8 17.8 16.4
220 200 180 160 140 120 100 80 60 40 20 0 ppm

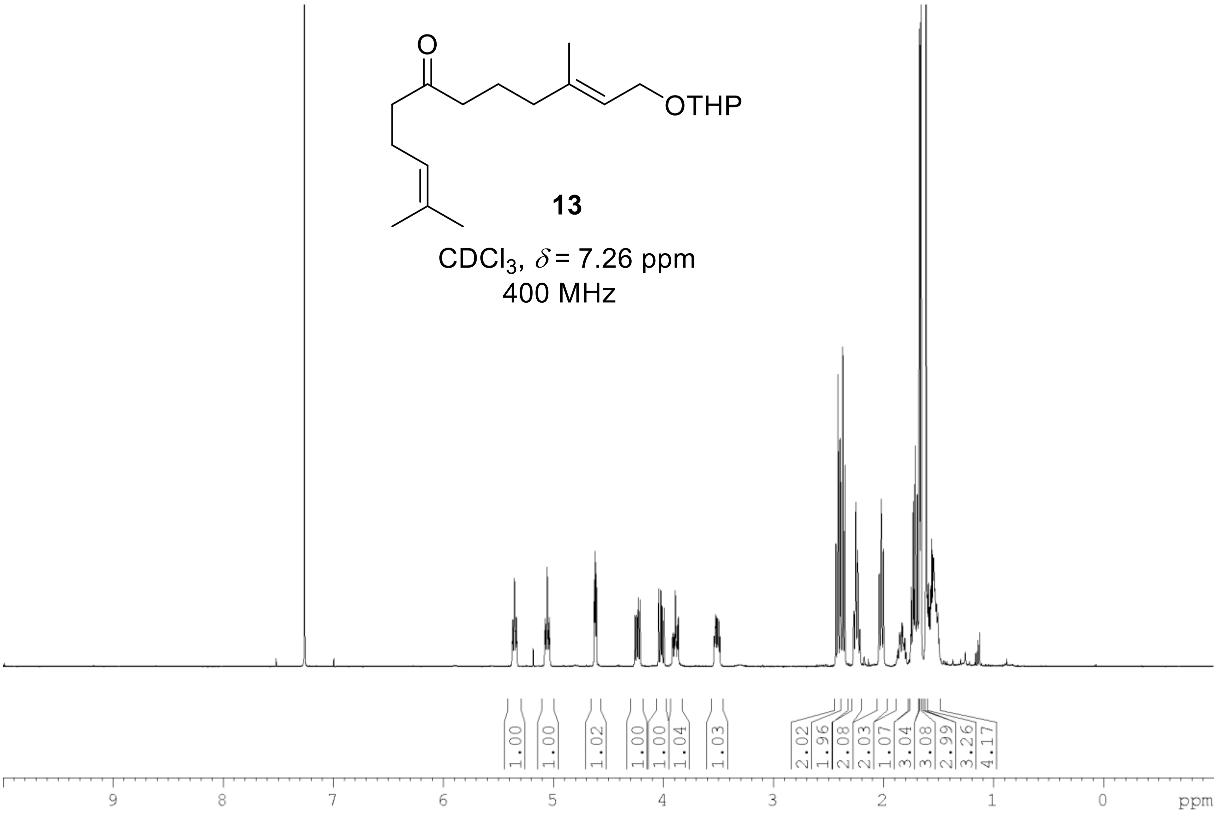

O
OTHP
13
CDCl3, δ = 7.26 ppm
400 MHz
1.00 1.00 1.02 1.00 1.00 1.04 1.03 2.02 1.96 2.08 2.03 1.07 3.04 3.08 2.99 3.26 4.17
9 8 7 6 5 4 3 2 1 0 ppm

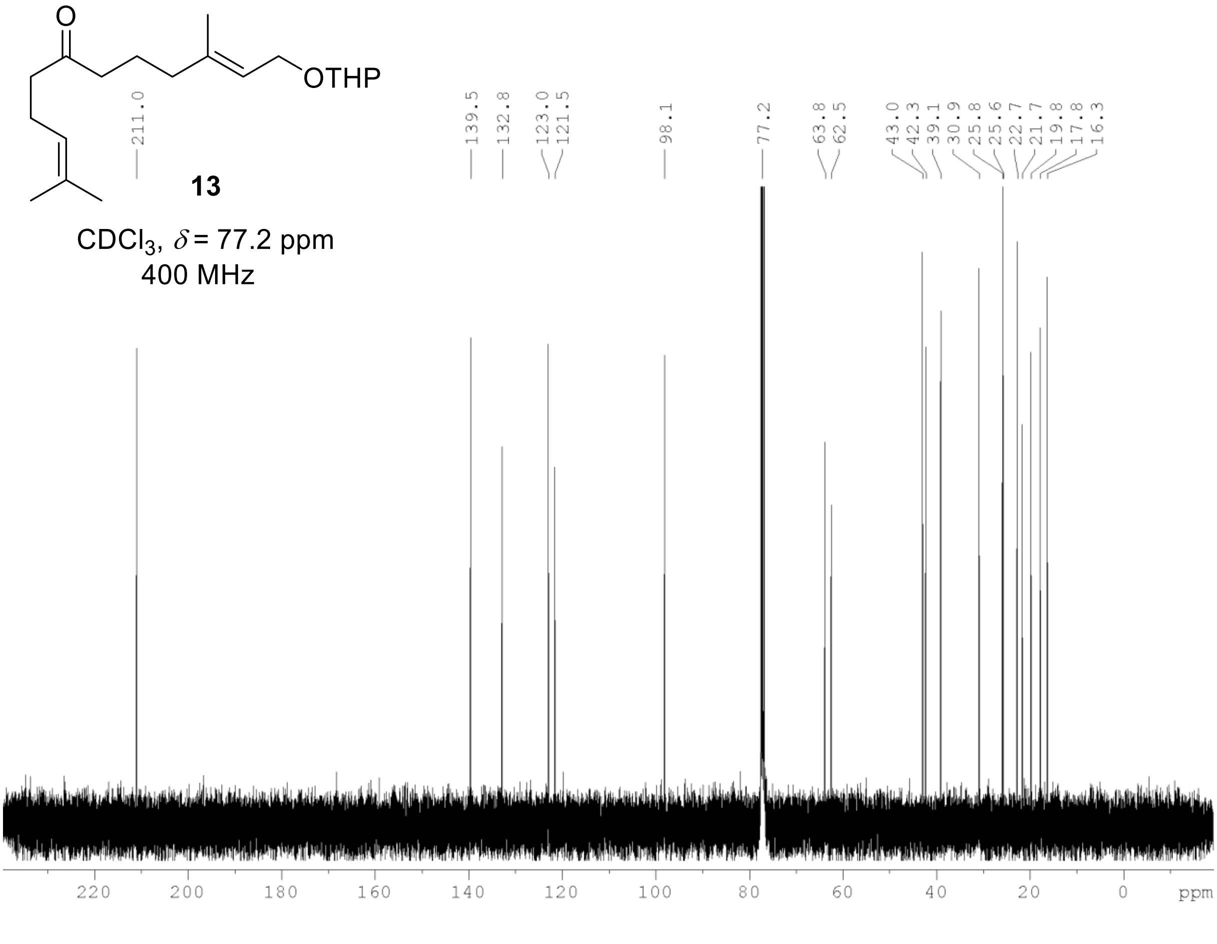

O
OTHP
13
CDCl3, δ = 77.2 ppm
400 MHz
211.0
139.5 132.8 123.0 121.5
98.1
77.2
63.8 62.5
43.0 42.3 39.1 30.9 25.8 25.6 22.7 21.7 19.8 17.8 16.3
220 200 180 160 140 120 100 80 60 40 20 0 ppm

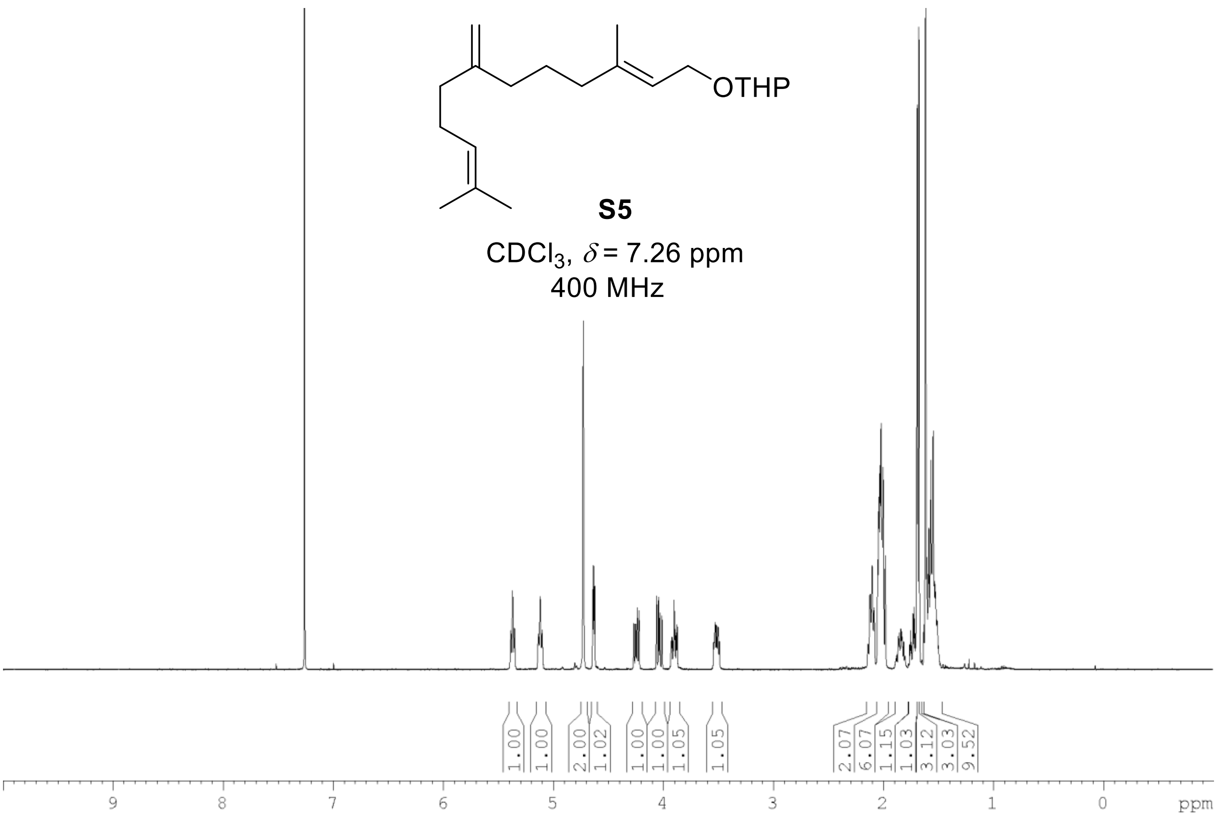
OTHP
S5
CDCl3, δ = 7.26 ppm
400 MHz
1.00
1.00
2.00
1.02
1.00
1.00
1.05
1.05
2.07
6.07
1.15
1.03
3.12
3.03
9.52
9
8
7
6
5
4
3
2
1
0
ppm

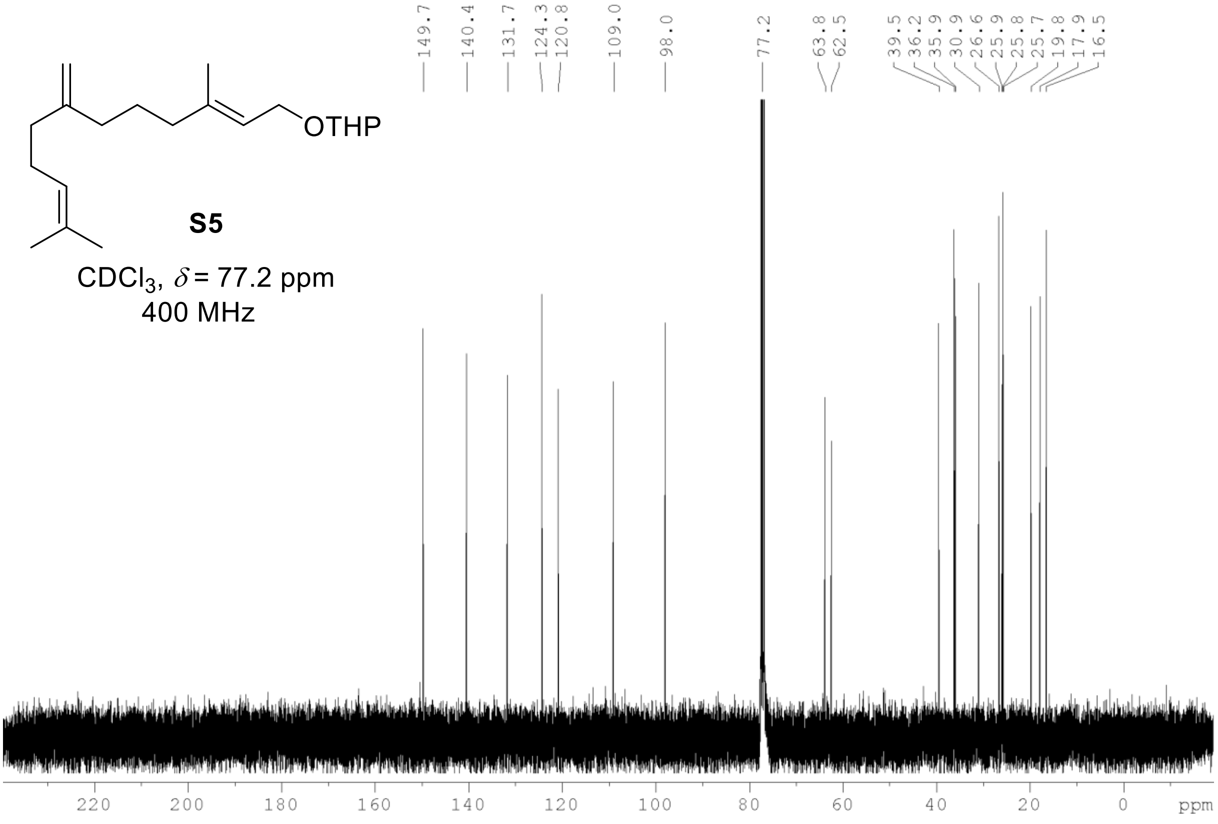
149.7
140.4
131.7
124.3
120.8
109.0
98.0
77.2
63.8
62.5
39.5
36.2
35.9
30.9
26.6
25.9
25.8
25.7
19.8
17.9
16.5
OTHP
S5
CDCl3, δ = 77.2 ppm
400 MHz
220
200
180
160
140
120
100
80
60
40
20
0
ppm

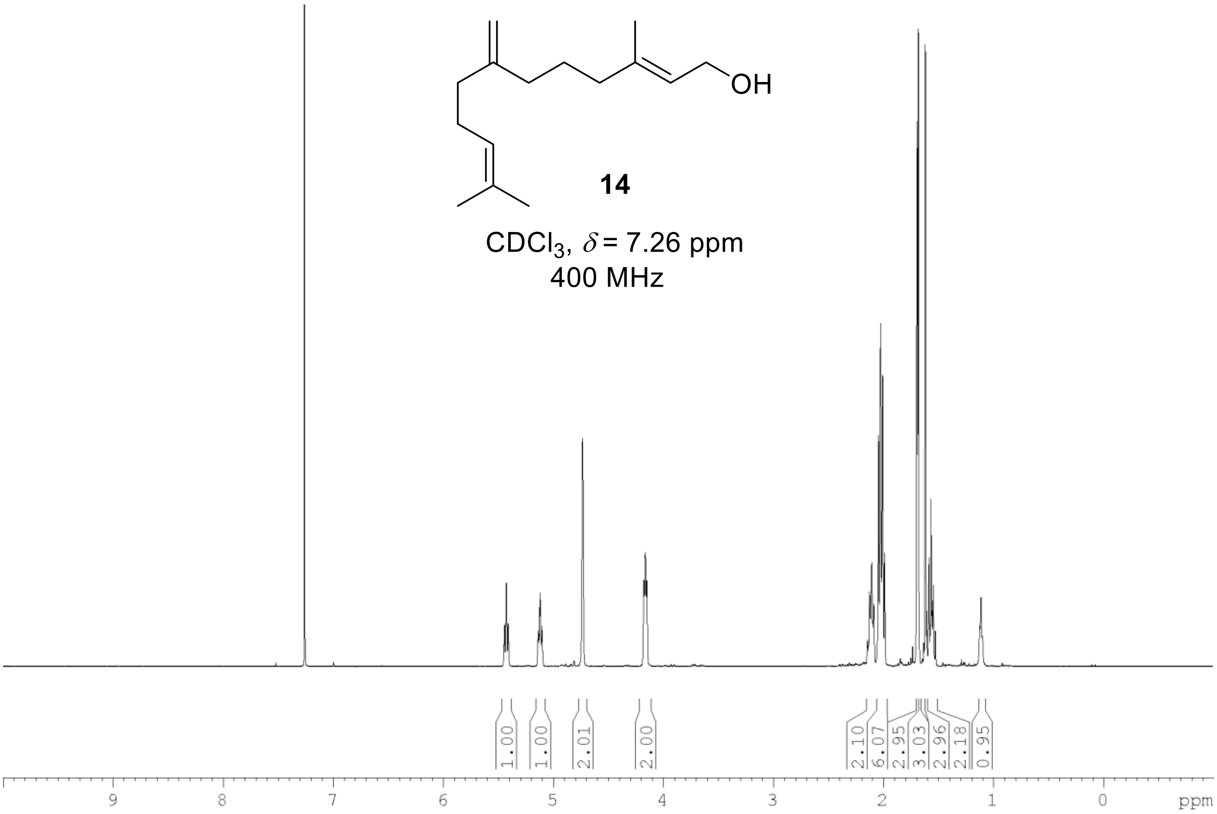
OH
14
CDCl3, δ = 7.26 ppm
400 MHz
1.00
1.00
2.01
2.00
2.10
6.07
2.95
3.03
2.96
2.18
0.95
9
8
7
6
5
4
3
2
1
0
ppm

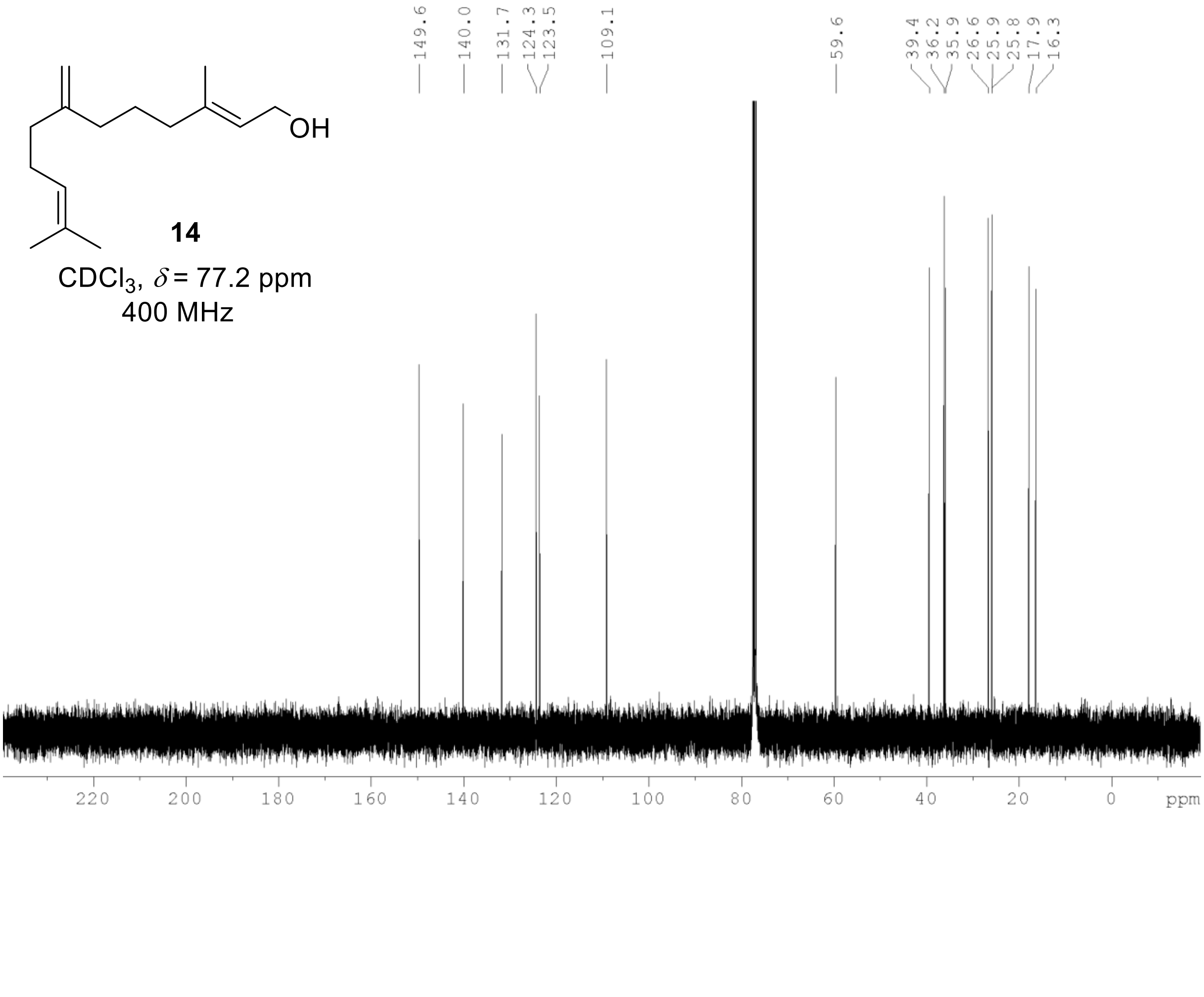
149.6
140.0
131.7
124.3
123.5
109.1
59.6
39.4
36.2
35.9
26.6
25.9
25.8
17.9
16.3
OH
14
CDCl3, δ = 77.2 ppm
400 MHz
220
200
180
160
140
120
100
80
60
40
20
0
ppm

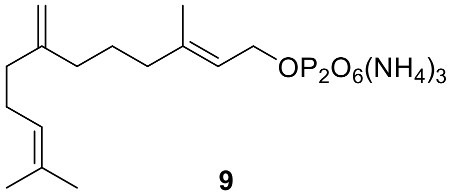

9

D_2O
400 MHz

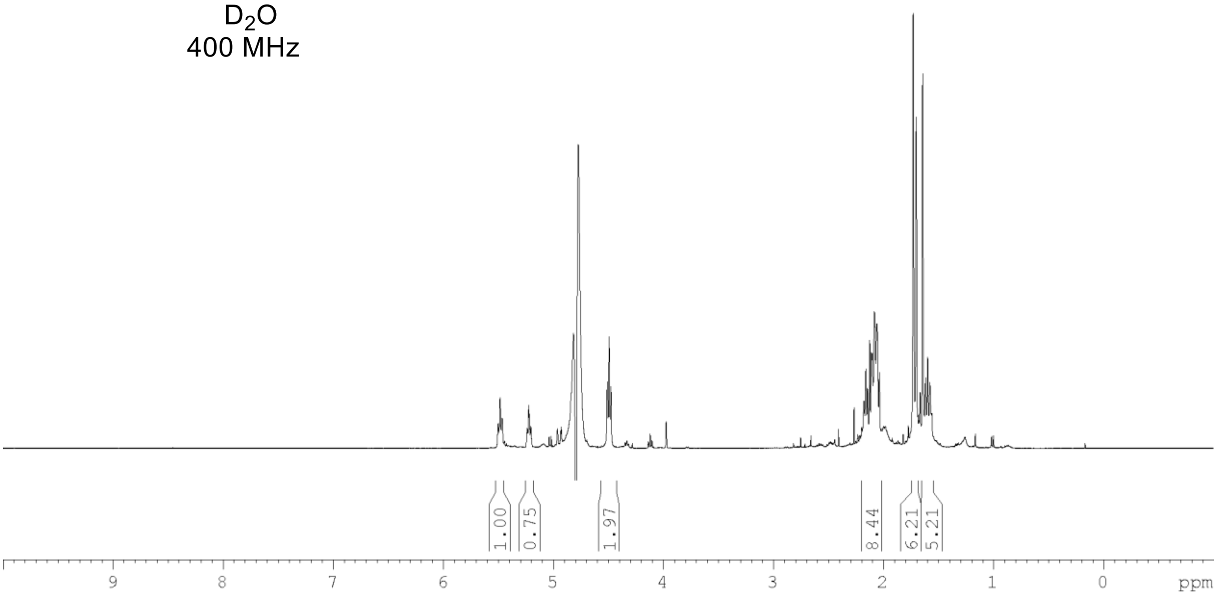

9

D_2O
400 MHz

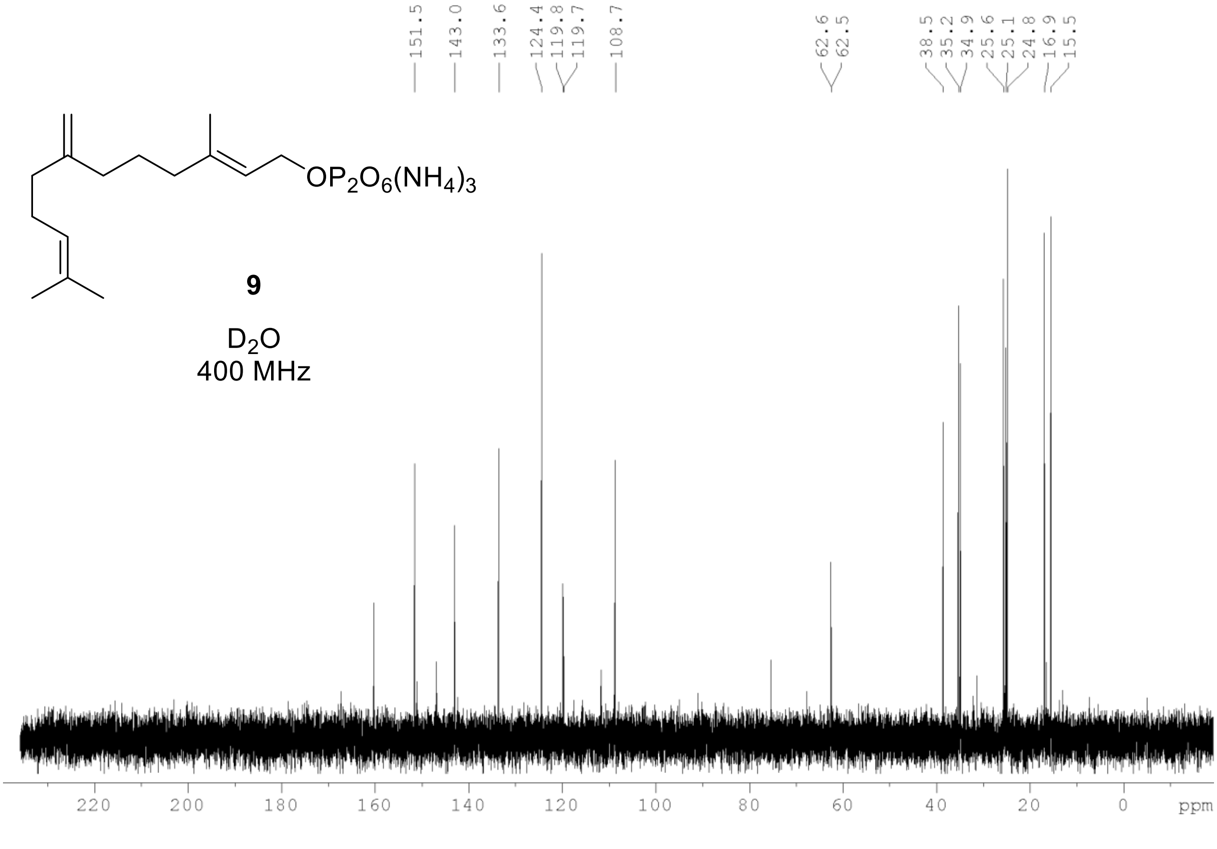

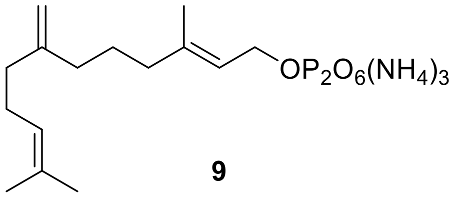

9

D_2O
400 MHz

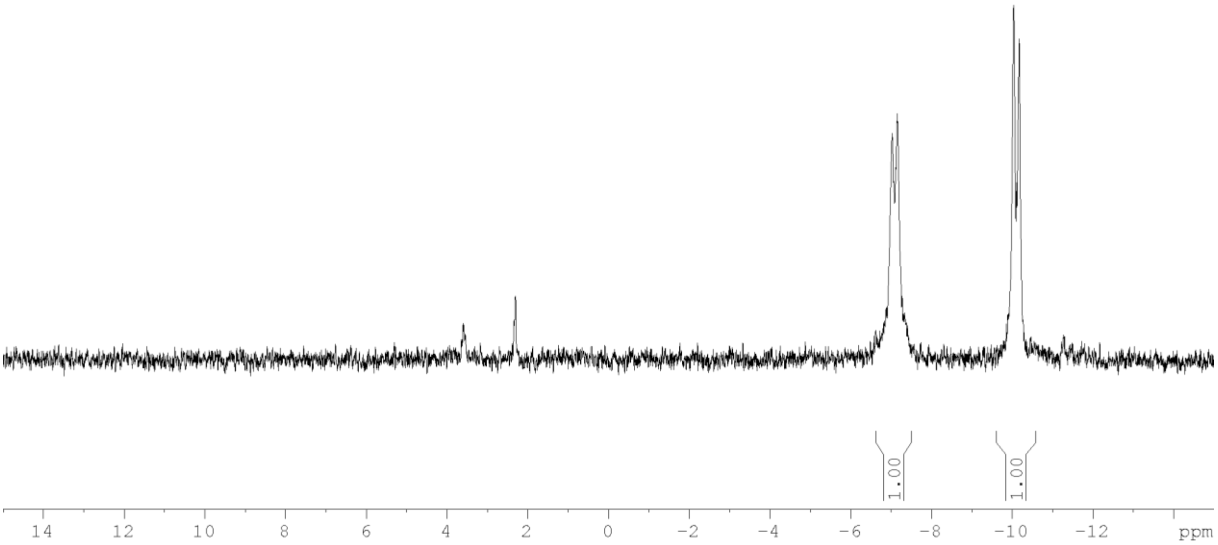

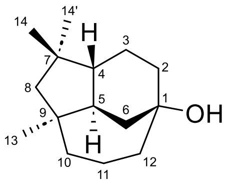

16

C_6D_6
600 MHz

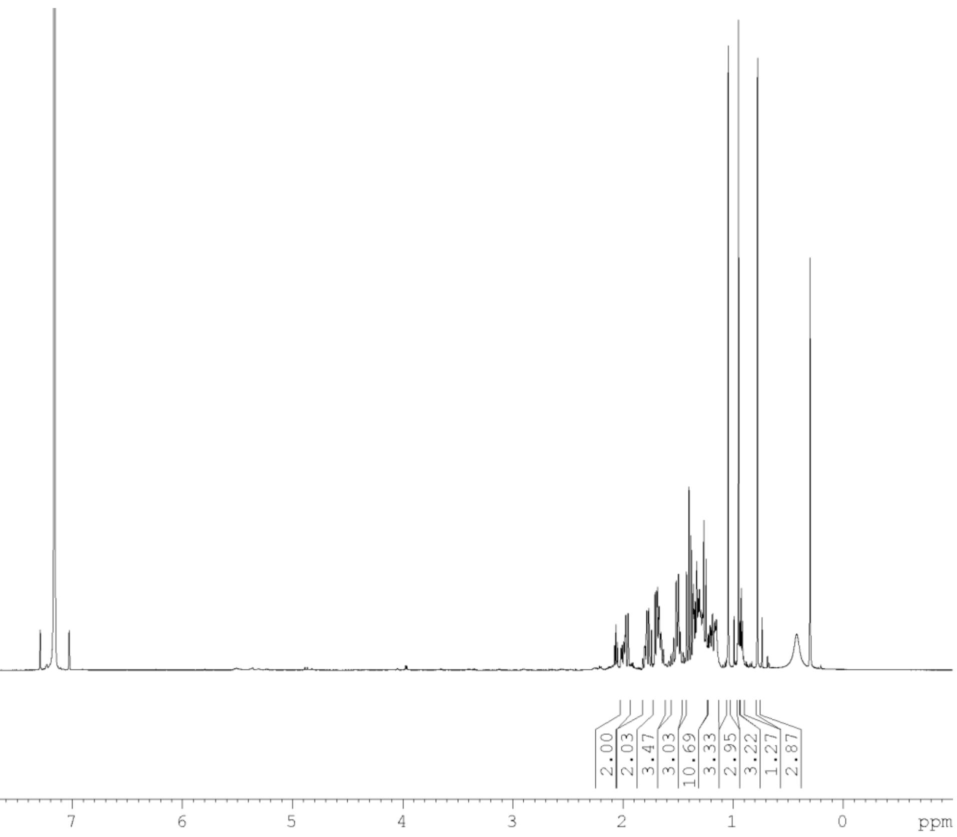

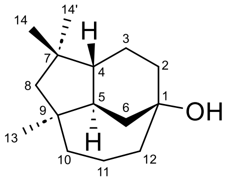

16

C_6D_6
150.1 MHz

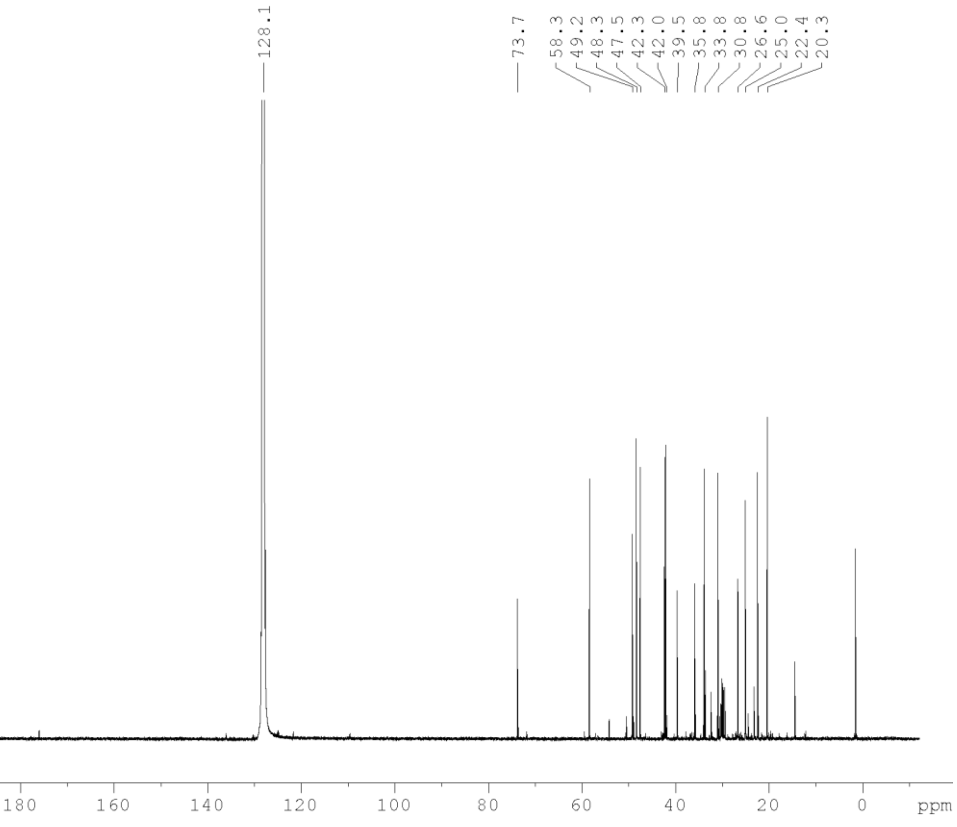

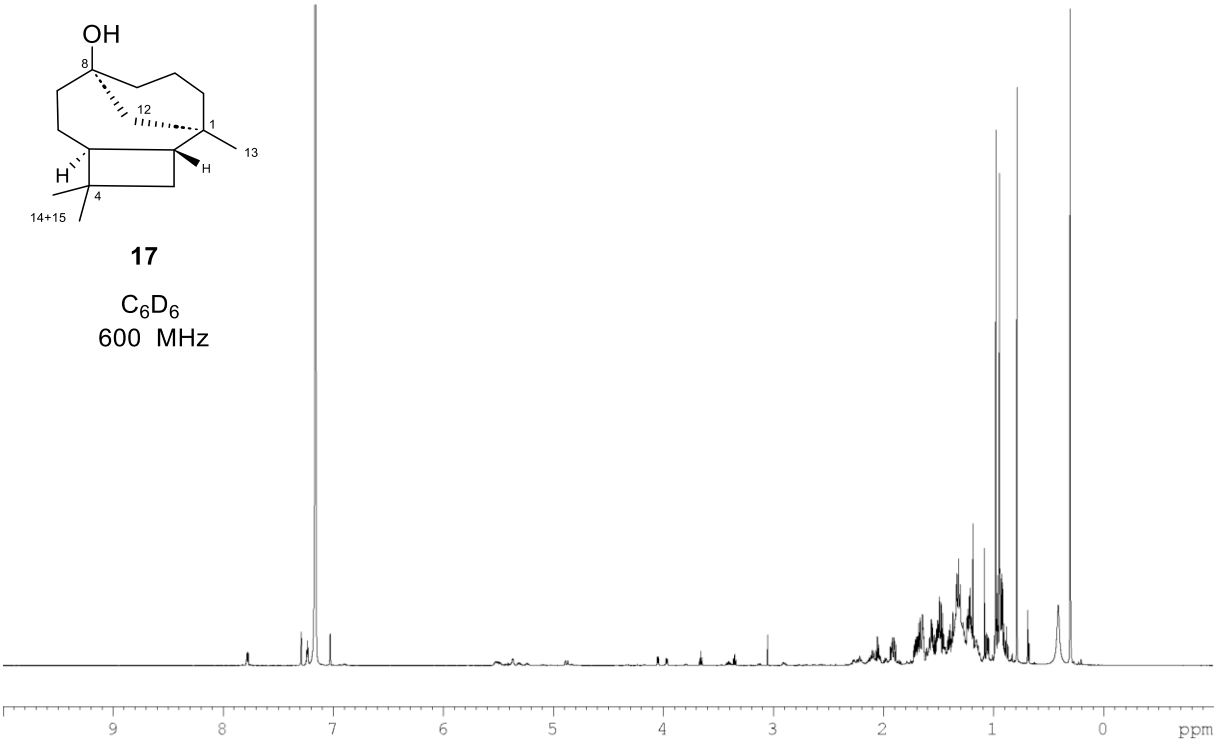
OH
8
12
1
13
H
H
4
14+15
17
C6D6
600 MHz
9
8
7
6
5
4
3
2
1
0
ppm

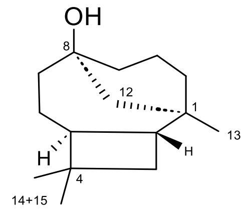

17

C_6D_6
150,1 MHz

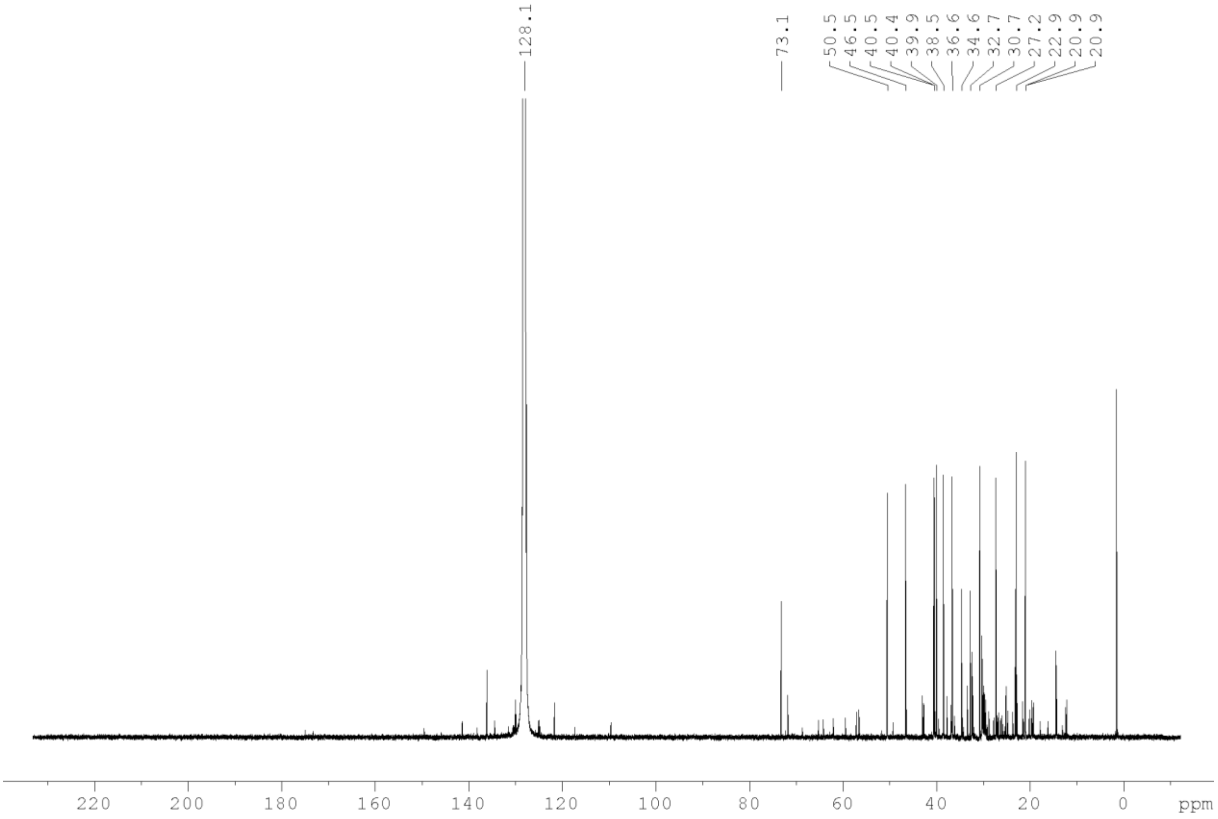

3. References (supporting information)

(1) Wang, C. M.; Hopson, R.; Lin, X.; Cane, D. E., Biosynthesis of the Sesquiterpene Botrydial in Botrytis cinerea. Mechanism and Stereochemistry of the Enzymatic Formation of Presilphiperfolan-8β-ol, *J. Am. Chem. Soc*. **2003**, *131*, 8360-8361.

(2) Nakano, C.; Horinouchi, S.; Ohnishi, Y., Characterization of a Novel Sesquiterpene Cyclase Involved in (+)-Caryolan-1-ol Biosynthesis in Streptomyces griseus*, *J. Biol. Chem.* **2011**, *286*, 27980-27987.

(3) Brandt, D.; Bellosta, V.; Cossy, J., Stereoselective Synthesis of Conjugated Trienols from Allylic Alcohols and 1-Iodo-1,3-dienes, *Org. Lett*. **2012**, *14*, 5594 – 5597.

(4) Armbrust, K. W.; Beaver, M. G.; Jamison, T. F., Rhodium-Catalyzed Endo-Selective Epoxide-Opening Cascades: Formal Synthesis of (−)-Brevisin, *J. Am. Chem. Soc.* **2015**, *137*, 6941 – 6946.

(5) Temple, K. J.; Wright, E. N.; Fierke, C. A.; Gibbs, R. A., Exploration of GGTase-I substrate requirements. Part2: Synthesis and biochemical analysis of novel saturated geranylgeranyl diphosphate analogs, *Bioorg. Med. Chem. Lett* **2016**, *26*, 3503 – 3507.

(6) Faraldos, J. A.; Grundy, D. J.; Cascon, O.; Leonie, S.; Kamp, M. W. v. d.; Allemann, R. K., Enzymatic synthesis of natural (+)-aristolochene from a non-natural substrate, *Chem. Commun.* **2016**, *52*, 14027 – 14030.
